# Biopsy Needle Integrated with Electrical Impedance Sensing Microelectrode Array towards Real-time Needle Guidance and Tissue Discrimination

**DOI:** 10.1038/s41598-017-18360-4

**Published:** 2018-01-10

**Authors:** Jaeho Park, Won-Mook Choi, Kyuyoung Kim, Won-Il Jeong, Joon-Beom Seo, Inkyu Park

**Affiliations:** 1 Department of Mechanical Engineering, Korea Advanced Institute of Science and Technology (KAIST), Daejeon, 305-701 Korea; 2 Laboratory of Liver Research, Graduate School of Medical Science and Engineering, Korea Advanced Institute of Science and Technology (KAIST), Daejeon, 305-701 Korea; 3 KI for NanoCentury & Mobile Sensor and IT Convergence (MOSAIC) Center, Korea Advanced Institute of Science and Technology (KAIST), Daejeon, 305-701 Korea; 4 Department of Radiology, Asan Medical Center, University of Ulsan College of Medicine, Seoul, Korea

## Abstract

A biopsy needle with electrical impedance sensor array based on stainless steel microelectrodes (EIS needle) was developed for real-time four electrode measurement and multi-spot sensing of tissues during the biopsy process. The sensor performance was characterized by using saline solutions with various concentrations, which proved accurate, stable and reliable electrical impedance measurement. The capability of impedance-based tissue sensing was verified by the conductivity measurement of agarose hydrogel based phantom mimicking cancer tissue. Furthermore, multi-spot impedance sensing during needle insertion was demonstrated using porcine meat with muscle and fat layers, which exhibited a clear discrimination between different types of tissues. Also, the electrical impedance difference between normal and fatty livers of mouse model was measured by the EIS needle. We could successfully demonstrate that the EIS needle can provide localized and accurate characterization of biological tissues at the needle tip.

## Introduction

**Figure 1:**
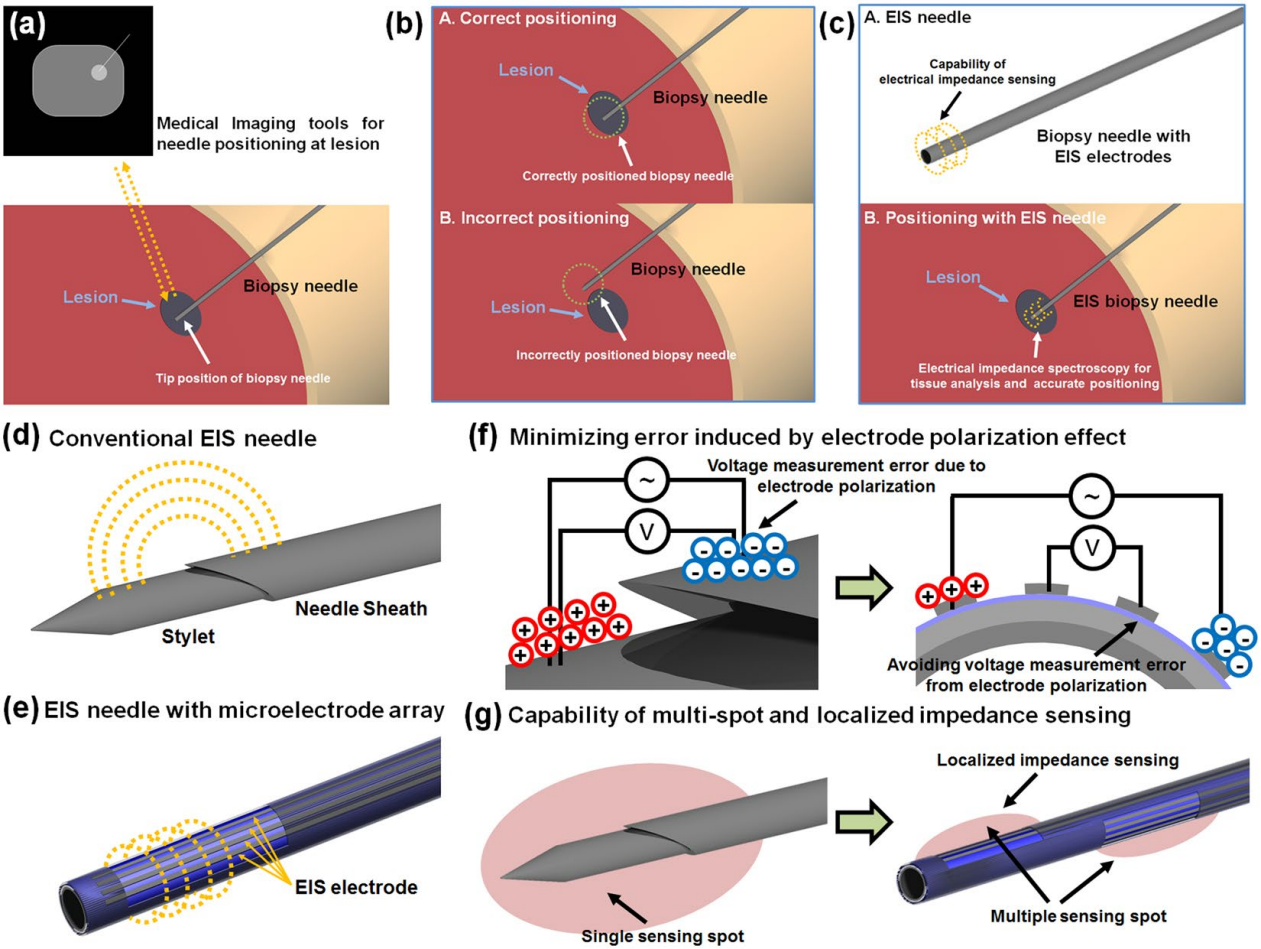
Impedance sensor integrated biopsy needle as a solution of tip positioning problem of conventional image-guided biopsy and conventional EIS needle vs. microelectrode array based EIS needle: (**a**) basic strategy to confirm the position of the biopsy needle tip; (**b**) potential problems about mis-positioning of the biopsy needle; (**c**) biopsy needle with electrical impedance sensor (EIS needle) for tumor discrimination and accurate needle positioning; (**d**) schematic image of conventional EIS needle for 2-electrode measurement; (**e**) schematic image of microelectrode array based EIS needle for 4-electrode measurement and multi-spot EIS; (**f**) measurement error induced by electrode polarization effect in conventional EIS with two electrodes and its resolution by using 4-electrode measurement; (**g**) multi-spot and localized sensing based on microelectrode array.

Image-guided biopsy is conducted in order to extract suspicious lesions from the patients for histological examination. In this procedure, correct tip positioning of biopsy needle is typically confirmed by using medical imaging tools such as ultrasound, computed tomography (CT) and magnetic resonance imaging (MRI)^1^ as shown in Fig. 1(a). Because the extracted tissue in the biopsy process is located right at the needle tip, locating the needle tip at the correct suspicious lesion is critically important as shown in Fig. 1(b). Although the positions of tumor and needle tip are distinguished by using medical imaging tools in many cases, the location of lesions in some organs is occasionally unclear, which increases the uncertainty and inaccuracy of the biopsy procedure. For example, the visualization of prostate cancers is often difficult with imaging tools, thereby radiologists extract tissues from 6 to 12 designated spots in the prostate^2^. In this case, an ultrasonic probe is utilized only for estimating the volume of prostate and locating the needle position^3^. Furthermore, since the imaging tools can only provide the location of lesion, it is difficult to obtain the detailed information of lesion such as type or stage of cancer before the histological assessment^2^.

Nowadays, the electrical impedance is considered as one of the most prominent indicators to discriminate the tissue types and to investigate the biological behaviors or changes of biological matters due to its high sensitivity, simple measurement method and real-time measurement capability^4^. Especially, the differences of electrical impedances between normal and cancerous tissues have been reported in various studies including the cases of lung, liver, breast, prostate cancer and etc. in both *in vivo* ^5,6^ and *ex vivo* measurements^7–13^.

Based on these advantages and previous studies on the electrical impedance measurement for tissue discrimination, biopsy needles with electrical impedance spectroscopy (EIS) capability to investigate the tissue properties near the needle tip have been developed by several researchers to overcome the above mentioned limitations of conventional image-guided biopsy process^14–17^ as shown in Fig. 1(c). In these works, the metal body of biopsy needle was directly used as electrodes as illustrated in Fig. 1(d). Typical biopsy needles used in the co-axial biopsy technique are composed of inner stylet and outer sheath in order to provide a space for containing the tissue cut by a sharp blade. In the above mentioned studies on EIS needles, an electrical insulation layer was made between inner stylet and outer sheath, and the needle tip was exposed to the external environment for electrical measurement. Therefore, the electrical properties of tissue near the needle tip could be investigated by measuring the electrical impedance between inner stylet and outer sheath. In contrary to the above mentioned EIS needle, Yun *et al*. reported the EIS needle with interdigitated electrodes for impedance measurement based on conventional semiconductor fabrication technique. They show the miniaturized interdigitated electrodes with width less than 400 μm which is applicable to needle with diameter of 700 μm (22-gauges)^17^. However, only 2-electrode measurement technique is possible in those configuration. When the electrical impedance of specimen with ions is measured, ions are attracted to the electrodes by electrostatic forces and an electric double layer generated by the ion attraction induces a local potential drop near the charged electrodes^18–20^. This phenomenon is called an electrode polarization (EP) effect and it causes considerable voltage measurement errors as shown in Fig. 1(f). It strongly occurs with 2 electrode measurement system in an alternative current with frequencies less than 100 kHz, where critical information on the biological tissues can be obtained by EIS due to the interfacial relaxation of biological tissues^4^. Because of this EP effect, the measurement results in the previous reports inevitably included significant errors especially in the low frequency regime. It has been generally known that the EP effects can be avoided by using 4-electrode measurement with two pairs of electrodes, in which one pair (outer electrodes) is used for the current injection and the other pair (inner electrodes) is used for the voltage measurement^18,19^. Above mentioned EIS needles not only suffered from errors induced by the EP effects, but also were not capable of simultaneous multi-spot measurement since multiple electrode array was not fabricated at various spots along the biopsy needle as shown in Fig. 1(g). Furthermore, the sensing area of the previously proposed EIS needle was in the range of several mm, and therefore the EIS needle could have low accuracy of tissue discrimination in heterogeneous tissue structures.

In this paper, we demonstrate a new type of biopsy needle with a capability of multi-point electrical impedance spectroscopy (EIS needle) by using microelectrode array fabricated on the surface of needle as shown in Fig. 1(e). Instead of utilizing the needle body as electrodes, we fabricated an array of microelectrodes with stainless steel microwires, and thus we could easily design the length and number of electrodes on-demand by attaching the stainless steel microwires on the surface of the biopsy needle. Therefore, for the first time, a biopsy needle with capabilities of 4-electrode measurement and multi-spot measurement with localized impedance sensing for accurate real-time tissue discrimination could be realized in this work as shown in Fig. 1(f,g). We demonstrated that our EIS needle could successfully function in 4-electrode measurement configuration with saline solutions and conduct real-time impedance sensing within an agarose hydrogel based phantom during needle insertion. Also the capability of multi-spot impedance sensing with a single EIS needle was verified with a real animal tissue. Furthermore, in order to verify the capability of our EIS needle in actual clinical situation, diagnostic performance was evaluated by computational analysis of local impedance measurement accuracy and by experimental analysis in which normal and fatty liver tissues in mouse model were distinguished with our EIS needle.

## Material and Methods

### Fabrication of EIS needle

**Figure 2:**
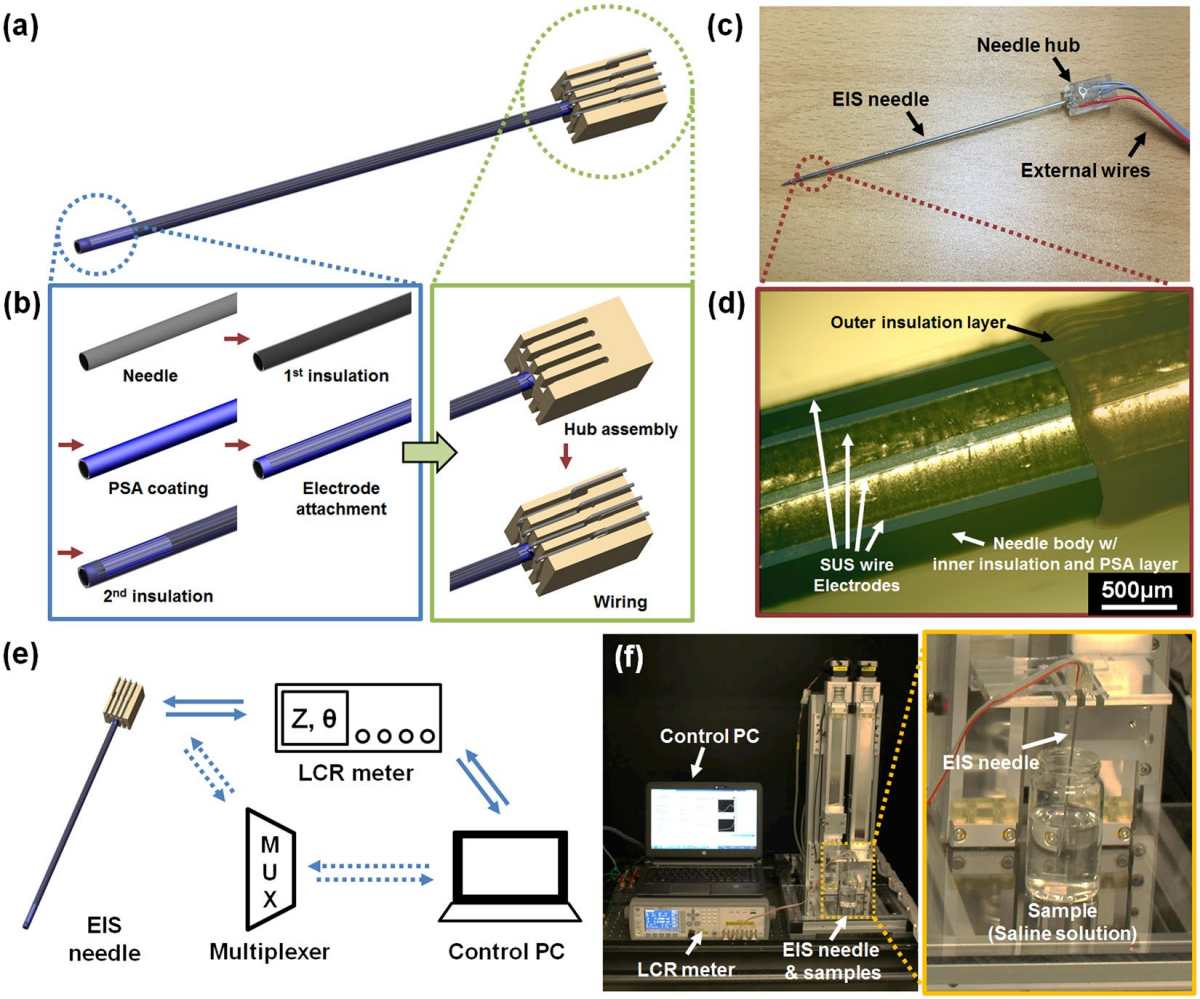
Fabrication process of EIS needle based on stainless steel micro-strips, photos of fabricated EIS needle and measurement system for EIS needle: (**a**) overall schematic of EIS needle; (**b**) fabrication process of microelectrode array on the surface of biopsy needle (blue box) and packaging to external connection (green box); (**c**) photograph image of fabricated EIS needle; (**d**) microscopic image at the sensing spot of EIS needle with four electrodes (electrode width = 110 μm); (**e**) connection diagram between EIS needle and measurement devices; EIS needle was connected to an LCR meter but a multiplexer unit was inserted in between EIS needle and LCR meter if multi-spot sensing was required; (**f**) photograph images of actual measurement setup of EIS needle; The EIS needle was fixed to a linear stage and allowed to move vertically in order to minimize human error during the measurement.

Figure 2(a,b) are schematic images illustrating the fabricated EIS needle and its fabrication process, respectively. Stainless steel 316 needles with diameter of 1.2 mm (gauge 18) and length of 15 cm (RF Medical, Korea) were used as biopsy needle bodies. First, the needle was encapsulated with a polyethylene terephthalate (PET) heat-shrink tube (Vention Medical, USA) in order to create an electrical insulation between the needle body and microelectrode array. The encapsulation was processed in a convection oven at 120 °C for 1 min. Then, a pressure sensitive adhesive (PSA) with a capability of instant fixation of attached electrodes was coated on the surface of insulated needle body. Other thermal or UV curing adhesives were not appropriate for fixing the electrodes because electrodes could not be immobilized on the surface of needle during the curing processes due to the small curvature radius of the needle. A biocompatible PSA (MD-7 4602, Dow Corning, USA) was diluted with ethyl acetate (Junsei Chemical, Japan) with a volume ratio of 1:1 in order to modulate the evaporation rate of solvent. The mixed PSA solution was first coated on a flat PET substrate by using a bar coater (OSP-10, OSP Korea, Korea) and then this was transferred to the needle surface by rolling the needle on the PET substrate. Afterwards, PSA was dried in an ambient air for 15 min. Then, the electrodes made of stainless steel 316LV flat strips (thickness = 25 μm, width = 110 μm and length = 10 cm; Fort Wayne Metals, USA) were prepared. (See Fig. S1 in the Supplementary Information) Before attaching the electrodes onto the surface of needle, 4 cm end of electrodes were electroplated with a 1 μm thick gold (Au) layer in order to make better adhesion to the external wire during soldering process. (See Fig. S2 in the Supplementary Information) Afterwards, the stainless steel electrodes were manually attached on the surface of needle. Due to the sticky nature of PSA layer, the electrodes could be fixed with a small amount of pressure. Although there existed small variation in the distances between electrodes, its effects on the EIS measurement could be calibrated by measuring the cell constant of the fabricated EIS sensor. The number and arrangement of electrodes could be varied depending on the number and locations of sensing spots. After the electrode fabrication, the second electrical insulation was made with PET heat-shrink tubes. In the second insulation, several spots on the heat-shrink tube were locally cut before the encapsulation in order to expose the sensing electrodes to the analyte in the external environment. Finally, customized needle hub made by 3D printing was attached at the end of needle and electrodes were connected to the external wire with a diameter of 0.312 mm (Eunsung Inc., Korea) by conventional soldering process. Figure 2(c) shows the photographic image of the fabricated EIS needle. As shown in the figure, the connection between the electrode and the external wire was fixed in the trench of needle hub with an epoxy glue. Magnified view of the electrodes connected on the needle hub is shown in Fig. S3 in the Supplementary Information. Also, the ends of external wires were finished with Bayonet Neill-Concelman (BNC) connectors for a connection to an LCR meter. The microscopic image of exposed region to the external environment is shown in Fig. 2(d). The stainless steel electrodes were attached in a good longitudinal alignment without delamination from the surface of needle. Furthermore, the electrodes were completely insulated with heat-shrink tube except for the open region for the electrical impedance measurement of the external environment.

### Measurement system configuration

Several equipment such as LCR meter, multiplexer unit and personal computer were used to measure and control the EIS system, and its overall layout is shown in Fig. 2(e). First, an LCR meter (E4980a, Keysight, USA) was utilized in order to give alternative current (AC) and to measure the voltage between electrodes to calculate the electrical impedance. Since the LCR meter was only compatible with single channel composed of four electrodes, the data acquisition unit (34970a, Keysight, USA) with multiplexer module (34904a, Keysignt, USA) was installed between the EIS needle and LCR meter to enable multi-spot EIS measurement. This overall measurement system was controlled by Labview^®^ in a personal computer, which could change the measurement parameters such as the frequency and amplitude of the AC current, selection of connected electrodes, etc.

Figure 2(f) illustrates the actual photograph image of the measurement system with EIS needle. The EIS needle was attached to the plate of vertical linear stage in order to minimize the measurement errors by avoiding vibrations from hands during the measurement. When the experiments with hydrogel phantom and biological sample were conducted, the experiment was performed in a manner similar to a conventional biopsy procedure. The conventional biopsy procedure proceeds in the order of “needle insertion”, “confirmation of needle position using medical imaging tools after needle stop”, “additional needle insertion”, and “tissue extraction after repeating the position correction process”. Therefore, we followed the similar order of “needle insertion”, “needle stop and impedance measurement”, “additional needle insertion”, and “needle stop and impedance measurement after reaching other type of tissue” in experiments. The insertion depth was estimated based on the length of needle outside the phantom or biological tissue because the insertion depth of the EIS needle cannot be accurately measured in optically opaque sample.

### Sample preparation

#### Preparation of saline solution

Saline solutions used for the characterization and calibration of sensor were made by mixing the deionized water and NaCl powder (7548–4400, Daejung Chemical & Metals, Korea). The target conductivity of saline solution at room temperature covering conductivity range of bio-tissue from 0.01 S/m to 1 S/m^21^ were 0.0103 S/m, 0.0257 S/m, 0.0616 S/m, 0.1537 S/m, 0.4084 S/m and 1.0115 S/m which corresponds to the concentration of 0.001 M, 0.0025 M, 0.006 M, 0.015 M, 0.04 M and 0.1 M in accordance with the relation between conductivity and concentration of NaCl solution^22^. However, due to the inaccuracy of manual preparation and other human errors, actual concentrations were slightly different from the target values. Thus, the actual conductivities of saline solutions at room temperature measured by the conductivity meter (Orion^TM^ Star A212, Thermo Fisher Scientific Inc., USA) were 0.0125 S/m, 0.0352 S/m, 0.0714 S/m, 0.2070 S/m, 0.4860 S/m and 1.0370 S/m, respectively.

#### Preparation of phantom model with normal and cancerous liver tissues

A phantom model was fabricated using commercial agarose hydrogel. The phantom consisted of inner core and outer covering agarose hydrogel that simulated the conductivity of cancerous and normal tissues in human liver, respectively. First, NaCl solutions were prepared in order to mimic the conductivities of normal and cancerous tissues in human liver (normal tissue: 0.03 S/m to 0.164 S/m and cancerous tissue: 0.166 S/m to 0.272 S/m) based on previous work of S. Laufer, *et al*.^10^. The conductivities of solutions mimicking normal and cancerous tissues were measured to be 0.0358 S/m and 0.2 S/m, respectively, using a commercial conductivity meter. In case of cancer mimicking solution, red ink was added in order to visually distinguish from surrounding normal tissue. Then, 1 wt% of agarose powder (016–13972, Daejung Chemical & Metals, Korea) was added into each solution, followed by heating to fully dissolve the agarose powder in the solution. The heated cancer mimicking solution was cooled down for gelation of solution and gelated tissue was cut into a 1.5 cm × 1.5 cm × 1.5 cm cube. The cancer mimicking cube was wrapped with Parafilm^®^ (Bemis, USA) in order to avoid diffusion of NaCl ions to the surrounding environment in the phantom. Then, heated agarose solution that mimics normal tissue was poured over cancer mimicking cube. Finally, cancer mimicking hydrogel was embedded within normal tissue mimicking hydrogel.

#### Preparation of porcine meat and fatty-liver mouse model

A porcine meat within one day after sacrifice was utilized for longitudinal EIS measurement. The meat had a clear boundary between fatty and muscular regions and was placed in a customized plastic container. As a sample for the mouse fatty liver experiment, male C57BL/6 wild-type (WT) mice purchased from the Jackson Laboratory (Bar Harbor, ME) were used. Four- to five-week old mice were fed with standard diet or high-fat diet (60% fat by calories; Harlan Teklad TD 06414, Madison, WI) for 12 weeks in a specific pathogen-free facility. At the end of 12-week standard diet or high-fat diet feeding, electrical impedance measurement was performed. Briefly, under inhalatory anesthesia using isoflurane, mice received a midline incision along the abdominal cavity to allow for the visualization of the liver. The EIS needle was inserted in the center of left lateral lobe to measure the electrical impedance. All mice received humane care according to the criteria outlined in the Guide for the Care and Use of Laboratory Animals provided by the National Institutes of Health (NIH), and all experimental procedures were approved by the Institutional Animal Care and Use Committee of KAIST.

### Computational analysis for impedance sensing range and performance evaluation of EIS needle based biopsy process

For computational analysis of tissue conductance measured by EIS needle, a commercial multi-physics simulation tool, COMSOL^®^ (COMSOL Inc., USA), was used. The conductance of tissue at the needle tip was calculated by changing the distance between the center of the EIS needle and the center of circular cancer tissue in a complex of normal and cancerous liver tissues as shown in Fig. 4(a). (See Supplementary Information about detailed model configuration for the computational analysis)

In order to calculate the sensing range of the EIS needle, we used commercial mathematical software, Matlab^®^ (Mathworks, USA). It was calculated based on 10% and 90% cutoff conductances, which are defined as 10% and 90% changes between conductances of normal and cancerous liver tissues with infinitely large diameter by the following equation (See Fig. S6 in Supplementary Information for detail):

1 $${G}_{{\rm{n}} \% {\rm{cutoff}}}={G}_{{{\rm{infinite}}}_{-}{\rm{normal}}}+(n\times 0.01)\cdot ({G}_{{{\rm{infinite}}}_{-}{\rm{cancer}}}-{G}_{{{\rm{infinite}}}_{-}{\rm{normal}}})$$

where *G*_*infinite_normal*_ and *G*_*infinite_cancer*_ are the conductances when the EIS needle is dipped in infinitely large normal or cancerous tissues, and *n* is the cutoff percentage. The 10% and 90% cutoff locations of EIS needle are obtained by the following criteria and used for the calculation of sensing ranges:

2 $${\rm{At}}\,x={x}_{10 \% {\rm{cutoff}}}\,:\,G(x)={G}_{{{\rm{infinite}}}_{-}{\rm{normal}}}+0.1\times [{G}_{{{\rm{infinite}}}_{-}{\rm{cancer}}}-{G}_{{{\rm{infinite}}}_{-}{\rm{normal}}}]$$

3 $${\rm{At}}\,x={x}_{90 \% {\rm{cutoff}}}\,:\,G(x)={G}_{{{\rm{infinite}}}_{-}{\rm{normal}}}+0.9\times [{G}_{{{\rm{infinite}}}_{-}{\rm{cancer}}}-{G}_{{{\rm{infinite}}}_{-}{\rm{normal}}}]$$

where *G(x)* is the EIS conductance at needle position x within the complex of normal and cancer tissues.

Monte-Carlo simulation was carried out in order to evaluate the diagnostic performance of the EIS needle based biopsy process using a commercial mathematical software, Matlab^®^ and conductance profiles along needle position that were calculated as explained above. (See Fig. S7 in Supplementary Information for detailed simulation process). The sensitivity and the specificity of EIS needle based biopsy process were calculated based on the result and below equations.

4 $${\rm{Sensitivity}}=\frac{{\rm{Number}}\,{\rm{of}}\,{\rm{true}}\,{\rm{positives}}}{{\rm{Number}}\,{\rm{of}}\,{\rm{true}}\,{\rm{positives}}+{\rm{Number}}\,{\rm{of}}\,{\rm{false}}\,{\rm{negatives}}}$$

5 $${\rm{Specificity}}=\frac{{\rm{Number}}\,{\rm{of}}\,{\rm{true}}\,{\rm{negatives}}}{{\rm{Number}}\,{\rm{of}}\,{\rm{true}}\,{\rm{negatives}}+{\rm{Number}}\,{\rm{of}}\,{\rm{false}}\,{\rm{positives}}}$$

The receiver operating characteristic curve (ROC curve) was plotted and area under ROC curve (AUC) was calculated in order to evaluate the performance of EIS needle based biopsy process.

## Results

### EIS sensor characterization

**Figure 3:**
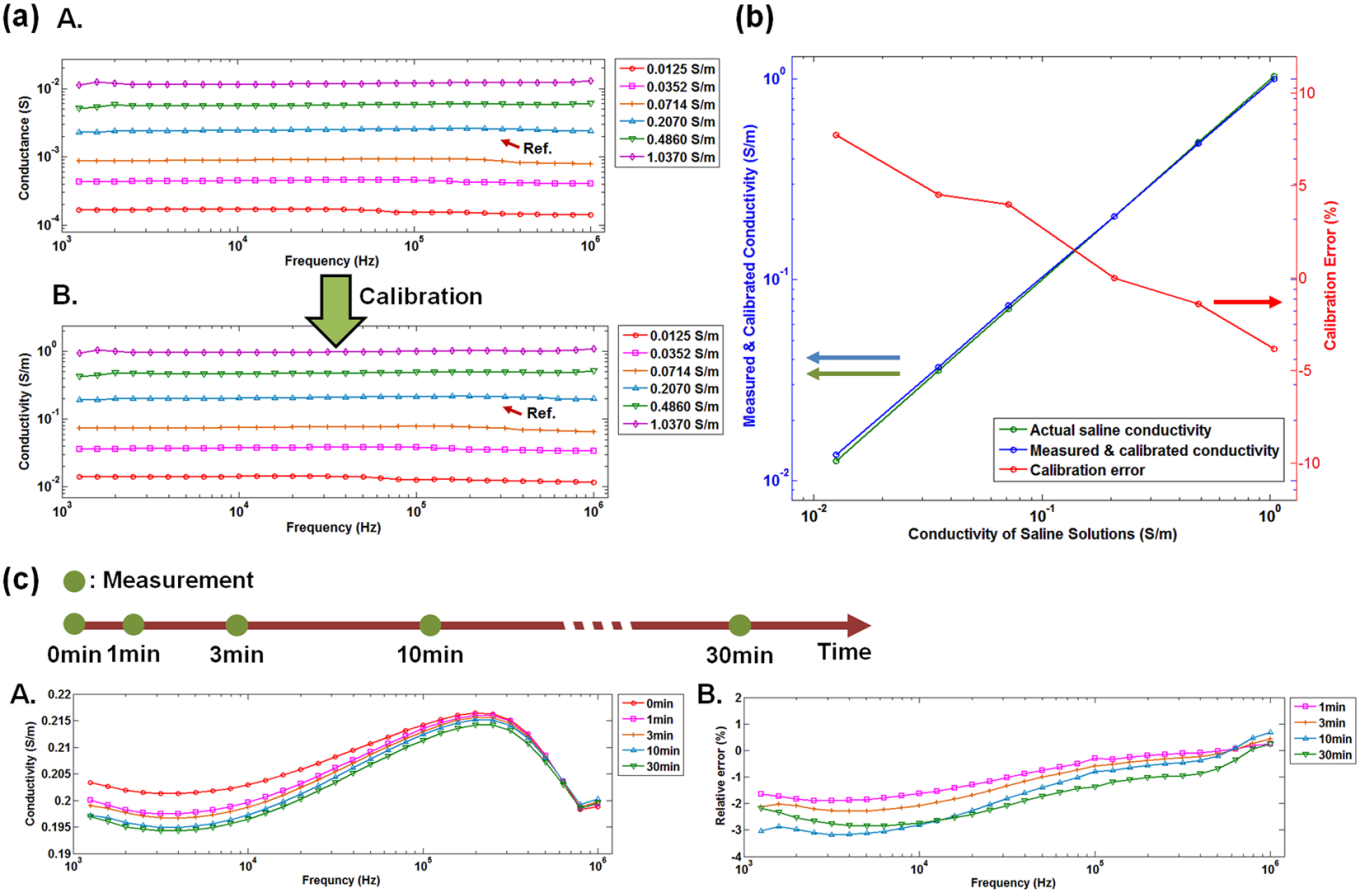
Electrical impedance sensing performance of EIS needle: (**a**) conductance measurement and conversion of conductance to conductivity: A. conductance measurement by changing frequencies from 1 kHz to 1 MHz, B. converted conductivity value using cell constant derived from reference solution (0.2070 S/m); (**b**) Calibration error after converting conductance to conductivity; relative errors (red line) between the conductivity measured by commercial conductivity meter (green line) and by the fabricated EIS needle (blue line) is shown; (**c**) stability test: A. change of measured conductivity of saline solution from 1 kHz to 1 MHz at 1, 3, 10 and 30 min after initial measurement, B. relative error from initial measurement.

We obtained the cell constant of fabricated EIS needle and characterized its calibration accuracy using saline solution with various conductivities. (See Supplementary Information for the definition and determination of the cell constant) The saline solution with a conductivity of 0.2070 S/m, which is within the range for the conductivity of human tissues^21^, was used as a reference to obtain the cell constant. The conductance of saline solution was measured using an LCR meter and the cell constant was calculated using measured conductance and conductivity of the saline solution. Then, calculated cell constant was utilized to convert the conductance of arbitrary saline solution into its conductivity, which is an intrinsic property of the solution. Figure 3(a) shows the measured conductance of saline solutions with various concentrations by changing the measurement frequency from 1 kHz to 1 MHz and their calibrated conductivities. The measured mean conductance of reference solution and standard deviation in the measurement frequency range were 2.465·10^−3^ S and 7.970·10^−5^ S, respectively. The calculated cell constant of fabricated EIS needle was 0.0119 m, which was obtained by dividing the measured mean conductance by the conductivity of reference solution. The conductance of other saline solutions with various concentrations were also measured and converted into the conductivity. The errors between actual and measured conductivities for different concentrations are shown in Fig. 3(b). Here, the calibration errors were obtained by the following formula:

6 $${\rm{Calibration}}\,{\rm{error}}\,( \% )=({\sigma }_{{\rm{mean}}}-{\sigma }_{{\rm{actual}}})/{\sigma }_{{\rm{actual}}}\times 100$$

where *σ*_*mean*_ is the mean conductivity from measured conductance for the frequency range from 1 kHz to 1 MHz and *σ*_*actual*_ is the conductivity measured by the commercial conductivity meter (Orion Star A212, Thermo Scientific, USA). All of the calibration errors in saline solutions with conductivities from 0.0125 S/m to 1.0370 S/m were within ±8%. These errors appear to be mainly induced from parasitic impedance of electrode arrangement on the surface of needle. Normally, error induced by parasitic impedance of probe was eliminated by using conventional open/short or open/short/load process. In case of microelectrode array, these approaches are not accurate because exact short state and adding loads to the end of microelectrodes could not be easily achieved. However, because the cell constants can be well fitted to a logarithmic function of conductivities of saline solutions as shown in Fig. S4 in the Supplementary Information, error could be also compensated by mathematically modeled cell constants obtained from saline solutions with various conductivities. (See Supplementary Information for detailed process of error compensation method) Therefore, we have confirmed that the fabricated EIS needle is capable of sensing the conductivity of ionic solutions with small errors. It should also be noted that the electrode polarization effect was not observed during the measurement with 4-electrode system: the conductance measured at 1 kHz was 86.7% of that measured at 1 MHz for a saline solution with a conductivity of 1.037 S/m. In case of 2-electrode measurement of saline solution with conductivity of 1.032 S/m as shown in Fig. S5(b), the conductance measured at 1 kHz was only 6.4% of that measured at 1 MHz, which was induced by the electrode polarization effect. (See Fig. S5 in the Supplementary Information)

The stability of EIS needle during long time measurement was also investigated as shown in Fig. 3(c). The EIS needle was soaked in the reference solution and its conductivity was repeatedly measured at 1 min, 3 min, 10 min and 30 min after the initial measurement. As time elapsed, measured conductivities were slightly shifted from the initial values. However, their relative changes from the initial measurement were within ±4%. This error may be attributed to partial change of the surface of stainless steel electrodes because pits can be generated on the surface in aqueous solution with chloride ions^23^. This degradation of electrodes results in a slow change of surface impedance of stainless steel^24^. Although there were small changes along the elapsed times, these deviations are insignificant because the needle insertion time is usually shorter than 30 min in the conventional image-guided biopsy process. The corrosion of electrodes may be critical for their biocompatibility in medical applications. However, the biocompatibility and corrosion resistance of stainless steel 316 have been extensively examined and it has already been widely used from simple surgical tools to medical implants^25^.

### Computational analysis for impedance sensing range and performance evaluation of EIS needle based biopsy process

**Figure 4:**
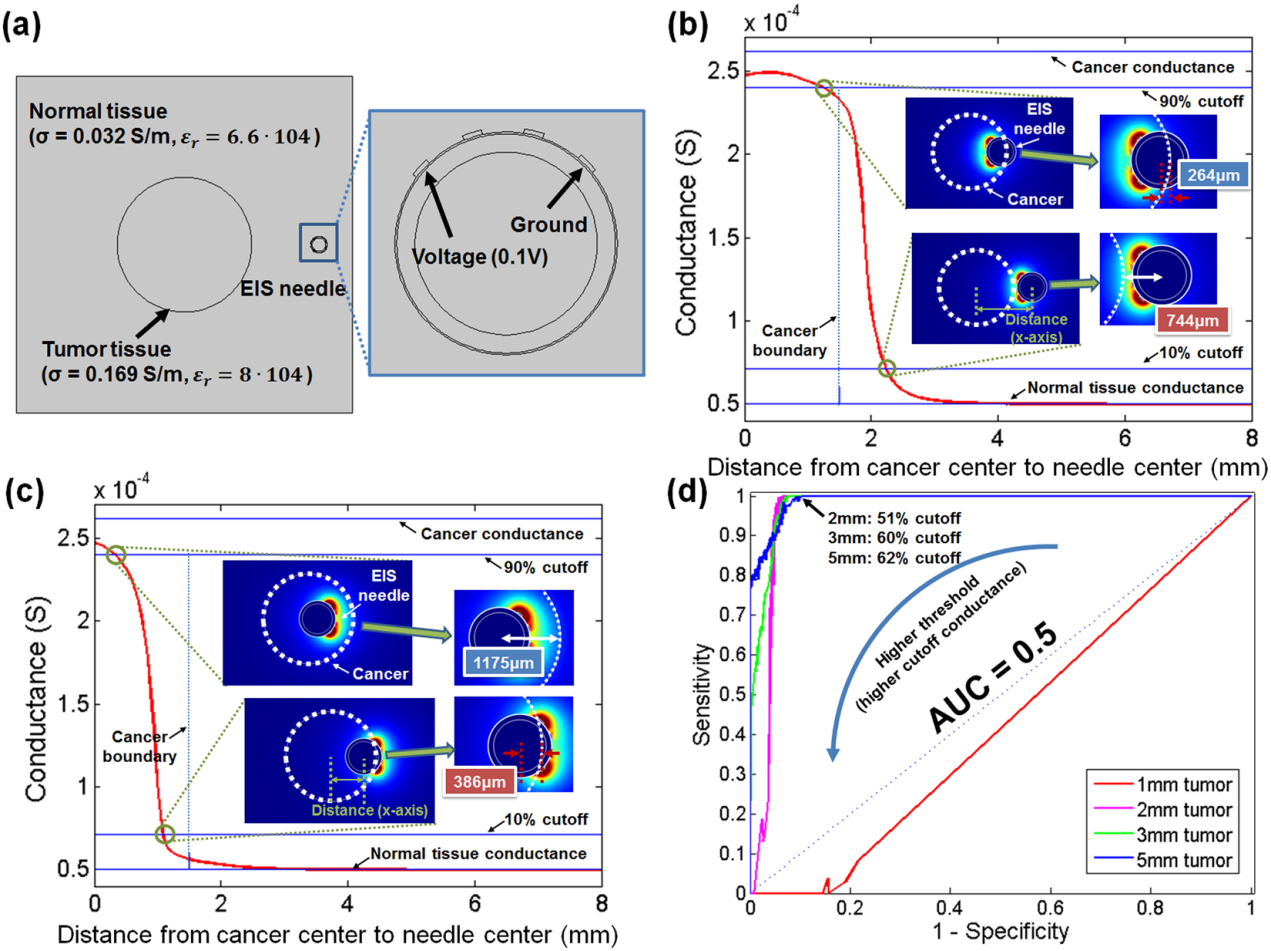
Computational analysis for impedance sensing range and performance evaluation of the EIS needle based biopsy process: (**a**) illustration of model for computational analysis including the EIS needle and the complex of normal and cancerous tissues; (**b**) the conductance profile of the EIS needle with electrodes facing towards cancer; (**c**) the conductance profile of the EIS needle with electrodes facing against cancer. The distances between the center of the EIS needle and cancer boundary for 10% and 90% cutoff positions are shown in the inset images of electrical field distribution; (**d**) computationally calculated receiver operating characteristic (ROC) curve of the EIS needle based biopsy process for cancer tissues with diameters of 1 mm, 2 mm, 3 mm, and 5 mm.

The sensing range of microelectrode array based EIS needle was analyzed by computational analysis. The conductance profile of cancer tissue with diameter of 3 mm is shown Fig. 4(b,c). (See Fig. S8 in Supplementary Information for sensing ranges for cancer tissues with diameters of 1 mm, 5 mm, and 10 mm) Fig. 4(b,c) are the conductance profiles when the electrodes of EIS needle face towards and oriented in the opposite direction from the cancer tissue, respectively. The position of the needle center and distance between needle center and cancer boundary at 10% and 90% cutoff are summarized in Table 1. If the 10% and 90% cutoff conductances are used as thresholds to distinguish between normal and cancer tissue, the interval between the 10% cutoff and the 90% cutoff is the region where the cancer and normal tissues cannot be distinguished, and these are 1.01 mm and 0.78 mm for the cancer-facing and reversely oriented EIS needles, respectively. If the EIS needle is placed in the above mentioned area, the EIS needle based cancer discrimination may fail. This area of diagnosis failure will be critical if the size of the cancer tissue is less than a few millimeters. In opposite, the probability of failure will decrease as the size of the cancer tissue increases.

**Table 1:** Calculated locations of EIS needle and distance between the center of EIS needle and cancer boundary for EIS electrodes facing towards cancer or in the opposite direction to the cancer tissue with diameter of 3 mm as shown in Fig. 4(b,c).

Cutoff percentage	EIS electrodes facing toward cancer		EIS electrodes facing in the opposite direction to cancer	
	Location of EIS needle	Distance between needle center and cancer boundary	Location of EIS needle	Distance between needle center and cancer boundary
10%	2.24 mm	0.74 mm	1.12 mm	0.39 mm
90%	1.24 mm	0.26 mm	0.325 mm	1.18 mm

Although the inaccuracy range of proposed EIS needle was less than 1 mm as explained above, it cannot be directly related to the actual diagnostic performance of the proposed EIS needle. In order to evaluate diagnostic performance of medical devices, receiver operating characteristic (ROC) curve has been usually employed as a facile tool for the easy visualization and reliable analysis^26,27^. A ROC curve is plotted based on the sensitivity and 1-specificity data obtained from actual diagnosis. However, because actual clinical simulation for the EIS needle is difficult to achieve, we conducted a computational method for the simulation of the biopsy process in order to calculate the sensitivity and specificity of the EIS needle that are defined in equation 4 and 5. Figure 4(d) is the ROC curve for the performance evaluation of the microelectrode based EIS needle. Here, sensitivity and 1-specificity are plotted for the cancer with diameter of 1 mm, 2 mm, 3 mm, and 5 mm by Monte-Carlo simulation. (See the Supplementary Information about detailed result of Monte-Carlo simulation using the customized result viewer program) If the size of cancer is 1 mm, the diameter of the biopsy needle is larger than that of cancer and the maximum measurable conductance is only a 41.9% cutoff conductance as shown in Fig. S8 (Supplementary Information). Therefore, when the size of the cancer is less than 1 mm, it is impossible to target the cancer using the EIS needle. In fact, it is shown that the area under ROC curve (AUC) of 1 mm cancer is less than 0.5, indicating that the diagnostic performance of EIS needle is totally worthless. However, as the size of the cancer increases to 2 mm, 3 mm, and 5 mm, AUC becomes 0.963, 0.983, and 0.990, respectively. These high AUC values reflect that the EIS needle based cancer targeting can be used as an excellent diagnostic tool. Here, ROC curve result was based on the assumption that the clinician can insert a biopsy needle with an accuracy of ±5 mm from center of cancer. Therefore, the position of the EIS needle was randomly selected within ±5 mm from the center of cancer for the simulation. However, if the clinician has more advanced biopsy skill and can insert the biopsy needle closer to the center of cancer, the usefulness of the proposed EIS needle may decrease. When the range of random insertion was decreased to ±4 mm and ±3 mm, the diagnostic performance of the EIS needle slightly decreased. However, all of AUCs of each ROC curves was still larger than 0.9 as shown in Fig. S9(b,c), which verifies excellent diagnostic performance of the EIS needle. (See the Supplementary Information).

### Tissue discrimination with EIS needle

#### Real-time EIS measurement in liver mimicking hydrogel phantom

**Figure 5:**
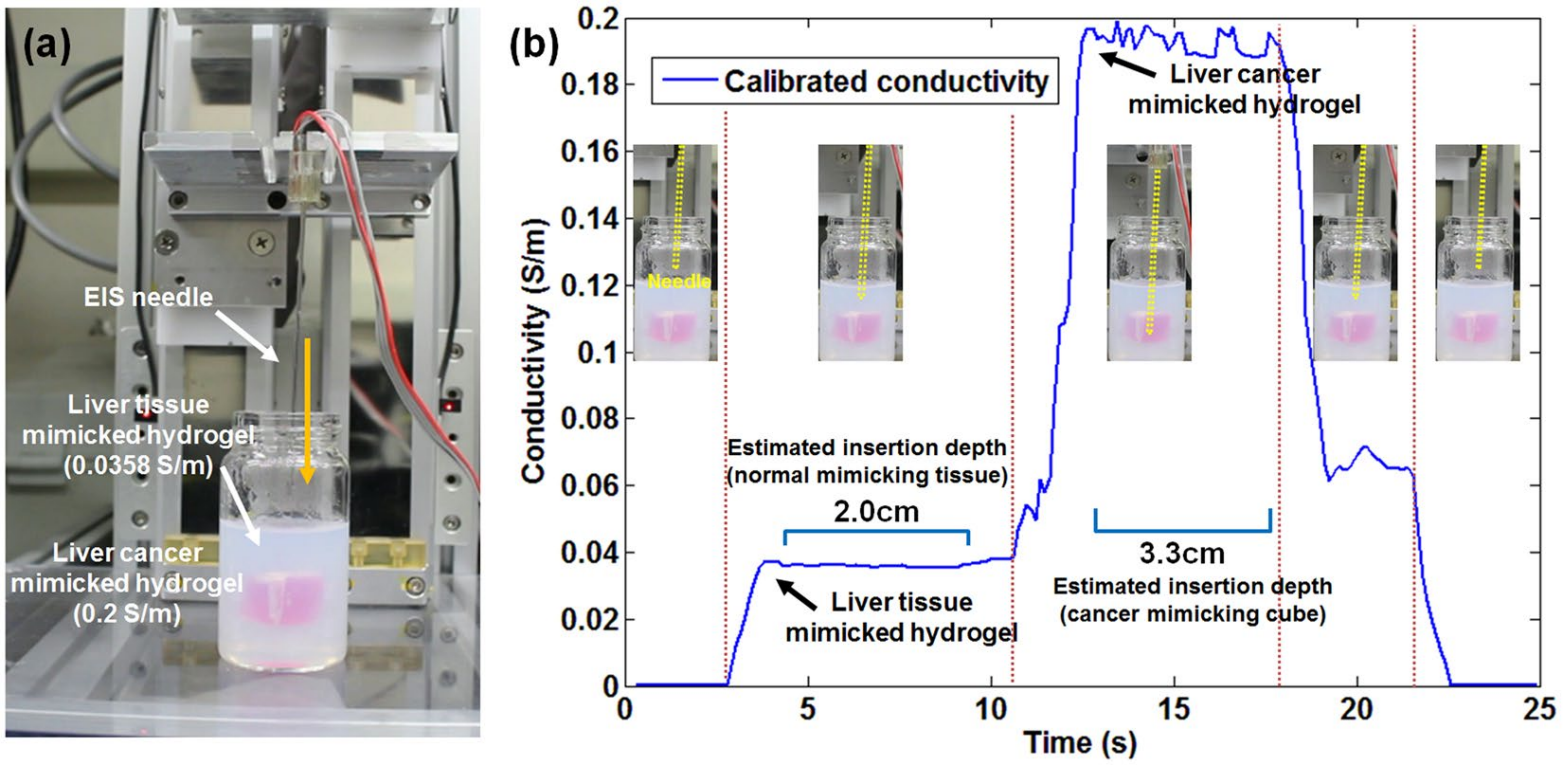
Real-time tissue discrimination during needle insertion in hydrogel phantom with cancer mimicking part (red color) surrounded by normal tissue mimicking part (white color): (**a**) photograph of experimental setup and hydrogel phantom; red cube (0.2 S/m) in the middle of phantom and surrounding hydrogel (0.036 S/m) mimic cancerous and normal liver tissues, respectively; (**b**) Real-time conductivity measurement at the needle tip during the needle insertion into the hydrogel phantom.

In order to verify the possibilities of tissue discrimination between normal and cancerous tissues, we conducted real-time EIS measurement during needle insertion into liver mimicking hydrogel phantom. A hydrogel phantom containing an embedded core with a conductivity of 0.20 S/m, which mimics a cancerous tissue, and surrounding matrix with a conductivity of 0.036 S/m, which corresponds to a normal tissue, was prepared as shown in Fig. 5(a). During the needle insertion through the phantom, conductance at the needle tip was measured real-time at a frequency of 100 kHz and conductance was calibrated by pre-calculated cell constant equation as illustrated in Fig S10. As shown in Fig. 5(b), when the EIS needle was inserted into the phantom, measured conductivity jumped to a mean vale of 0.036 S/m (See the Supplementary Video). When the needle was inserted further into the cancerous part, the conductivity at the needle tip was immediately increased to a mean value of 0.193 S/m. The relative difference between cancerous and normal parts was measured as 5.33 times by EIS needle, while the conductivity meter gave 5.59 times of relative difference. Therefore, we could verify that the fabricated EIS needle can measure the electrical conductivity and distinguish between normal and cancerous tissues real-time during needle insertion. When the needle was retreated to the normal part, the conductivity was dropped to a mean value of 0.0663 S/m. This is higher than 0.036 S/m that was measured before insertion into cancerous part, and this appears to be due to the leakage of ions from high concentration zone (cancerous part) to low concentration zone (normal part) through the hole made by the needle insertion.

#### Longitudinal porcine tissue discrimination with EIS needle

**Figure 6:**
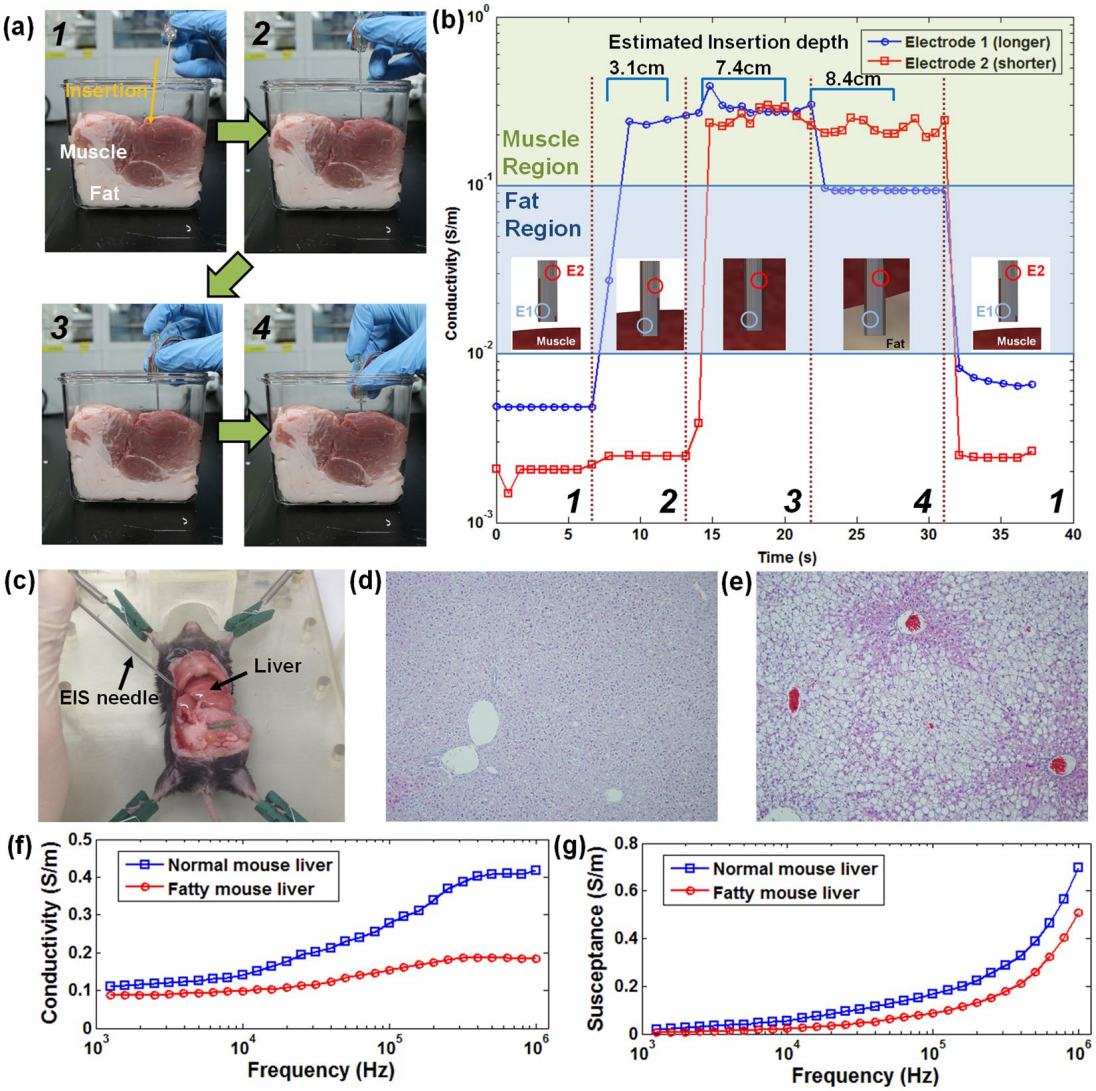
Multi-spot sensing for real-time tissue discrimination with porcine meat and EIS needle measurement of fatty liver mouse model: (**a**) photograph image of sequential needle insertion into porcine meat and schematic image showing the change of tissues in contact with each set of electrodes; (**b**) Measured conductivity at each set of electrodes during needle insertion; sensing region of electrode 1 (blue line) was at the end of needle while electrode 2 (red line) was 1 cm shorter than electrode 1. The conductivity range of muscle (green box) and fat (blue box) was based on Gabriel *et al*.’s work^21,28^. The italic numbers correspond with the sequences illustrated in (**a**); (**c**) photograph image of needle insertion experiment into the mouse liver; Histological images of (**d**) normal liver and (**e**) fatty liver of mouse; (**f**) conductivity and (**g**) susceptance of normal and fatty mouse livers from 1 kHz to 1 MHz using EIS needle.

We demonstrated that the EIS needle with multiple sets of four electrode array can simultaneously measure the electrical properties of biological tissues at several spots of the needle. Two sets of four electrode array were separated by 1 cm along the needle in order to obtain the electrical impedance at two dissimilar points along the path of the needle insertion. As an example specimen, a porcine meat containing both muscle and fat layers was used. It is generally known that typical conductivity ranges of muscle and fat are 0.1–1 S/m and 0.01–0.1 S/m, respectively^21,28^, and these values were used as references to identify the types of tissue near the electrode arrays. The needle was inserted manually into porcine meat in order to simulate a conventional biopsy procedure, which is performed manually. When the EIS needle was inserted into the porcine meat, the tissue in contact with each set of four electrode array were changed to either muscle or fat as shown in the Fig. 6(a). By indicating the differences in the impedance at different sets of electrodes, we could recognize the boundary between muscle and fat tissues and therefore the position of the biopsy needle tip could be estimated.

Figure 6(b) shows the real-time changes of electrical conductivity measured by each set of electrode array at a frequency of 1 kHz. (See the Supplementary Video) When the EIS needle tip was inserted slightly into the porcine meat, the longer electrode array (electrode 1; E1) first contacted with the muscle tissue, from which a conductivity of 0.26 S/m was measured. As the EIS needle was further inserted, the shorter electrode array (electrode 2; E2) also made a contact with the muscle tissue and therefore its measured conductivity was almost the same as that of electrode 1. However, when the needle was further inserted so that the needle tip reached the fat layer, the conductivity at electrode 1 suddenly dropped to 0.093 S/m while the conductivity at electrode 2 was still maintained above 0.2 S/m which was corresponded to muscle conductivity. Therefore, we could confirm that the EIS needle tip is able to detect its penetration from the muscle to the fatty tissue.

#### Comparison of EIS spectra between normal and fatty liver with EIS needle

In order to verify the capability of EIS needle to discriminate the normal and abnormal tissues in the same organ, we measured the complex admittance and impedance of the normal and fatty mouse livers using the fabricated EIS needle. Fatty liver is often considered to be the initial event in the pathogenesis of various liver diseases including nonalcoholic fatty liver disease (NAFLD) and alcoholic fatty liver disease (AFLD). Increased fat uptake and triglyceride deposition in hepatocytes is the key point, which causes NAFLD^29^. Previous studies have shown that the fatty liver tissues have higher electrical impedance than the normal liver tissues. The mechanism of impedance increase in the low frequency regime (100 Hz–100 kHz) is known as the expansion of cell size due to the accumulation of lipid inside cells, which makes the extracellular space smaller and reduces path for electrical current, particularly in the low frequency range. On the other hand, in the high frequency regime (>100 kHz), the current passing through the intracellular matrix becomes significant. Since the fat has higher impedance than other intracellular substances, the fat intruded into the cells increases the impedance of cells. Therefore, the impedance of the tissue is also increased in the high frequency regime^30^.

As shown in Fig. 6(c), the EIS needle was inserted into the liver and its impedance spectrum was investigated in the frequency range of 1 kHz to 1 MHz. Figure 6(f,g) shows the measured conductivity and susceptance values of normal and fatty mouse livers, which are summarized in Table 2. (See also Fig. S11 and Table S3 in Supplementary Information for the values in form of impedance and phase) Although the absolute values of electrical impedance of normal and fatty livers were different from previously reported data below 100 kHz of frequency^30^, the trend of impedance difference between normal and fatty livers was similar. In order to verify the histological difference between normal and fatty livers, the micro-structures of normal and fatty liver tissues were investigated as shown in Fig. 6(d,e), respectively. The liver in pair-fed group did not show any histological abnormalities with clear architecture including portal area and central vein. On the contrary, the high-fat diet fed mice clearly exhibited the macrovesicular and microvesicular fat accumulation inside the hepatocytes and the ballooning degeneration of hepatocytes as compared to the pair-fed group. This swelling of cells caused narrower electrical current path between the cells and induced relatively higher impedance in fatty liver model especially at low frequency region as explained in Parramon *et al*.’s work^30^. Therefore, we could confirm a good correlation between the differences in the electrical impedances of normal and fatty livers, and their histological differences.

**Table 2:** Measured values of conductivity and susceptancee of normal and fatty mouse livers at frequencies of 1 kHz, 10 kHz, 100 kHz and 1 MHz.

	Parameters	1 kHz	10 kHz	100 kHz	1 MHz
Normal mouse liver	Conductivity (S/m)	0.1095	0.1398	0.2947	0.4177
	Susceptance (S/m)	0.0199	0.0545	0.1669	0.6958
Fatty mouse liver	Conductivity (S/m)	0.0864	0.0989	0.1545	0.1842
	Susceptance (S/m)	0.0053	0.0227	0.0878	0.5086

## Discussion

In the present work, the microelectrode array was patterned on the surface of biopsy needle with diameter of 1.2 mm in order to solve the above mentioned limitations of the conventional biopsy needle with EIS sensing capability. The major advantage of this method is that the number, shape and length of electrodes can be freely designed by patterning the microelectrode array. The advantages from this novel approach are as follows.

Firstly, a 4-electrode measurement which cannot be realized with the conventional EIS biopsy needle can be used. The 4-electrode method has been commonly used for the impedance measurement of biological tissues in order to reduce the measurement error from electrode polarization effect^18^. However, the application of 4-electrode method to surgical instruments having small surface area is very difficult because micro-scaled EIS sensor should be integrated in the small area, thus the biopsy needle body has been used as an electrode itself in the previous reports^16^. In this report, the microelectrode array based EIS needle could reduce error due to electrode polarization effect as shown in Fig. 3(a) where the conductivity of saline solution is measured to be constant from 1 kHz to 1 MHz of measurement frequencies. Therefore, it can be concluded that the microelectrode array based EIS needle can perform EIS accurately in biological tissues where ionic current is dominant and electrode polarization effect may severely occur in a two electrode system.

Secondly, localized impedance sensing can be conducted using micro-scale electrodes while the previously reported EIS needles showed sensing ranges in the millimeter scale. The localized sensing area was analyzed by computational analysis as shown in Fig. 4(b) and (c), which proves the accuracy improvement in heterogeneous tissue by measuring the electrical characteristics of only the tissue directly in contact with the EIS sensor electrode array. In addition, the diagnostic performance of the microelectrode based EIS needle was computationally evaluated by using Monte-Carlo method and plotting ROC curves. As a result, it was confirmed that a diagnostic performances was sufficiently accurate for a lesion with diameter larger than 3 mm (AUC > 0.95) and this is because of above mentioned localized impedance sensing characteristics. According to previous clinical studies, in case of cancer size less than 1.5 cm, diagnostic accuracy of biopsy process is significantly reduced owing to practical problems on clinicians and relatively low spatial resolution of conventional medical tools (eg. 0.5 mm for computed tomography)^31,32^. Therefore, it is expected that the microelectrode array based EIS biopsy needle can provide more accurate positioning of biopsy needle with respect to the cancer tissue and therefore improve the diagnostic accuracy of biopsy for small-sized cancer tissues.

Thirdly, impedance measurements at several points around the biopsy needle can be simultaneously conducted. In the previous reports, impedance measurement could be performed only at the tip of the biopsy needle. However, in this work, the impedance measurement could be performed at various points around biopsy needle because the number, length and arrangement of the electrodes can be freely designed by our approach. As shown in Fig. 6(a,b), it was confirmed that the tissue distribution along the biopsy needle can be monitored in real-time using two sets of EIS electrode arrays which were placed at the needle tip and 1 cm apart from the needle tip. Based on this multi-site measurement, more accurate biopsy can be enabled by informing the clinician about the tissue distribution along the needle, especially in the case of complicated and heterogeneous tissue structure.

Finally, in terms of biocompatibility and fabrication cost, all materials used in the fabrication including electrode materials (stainless steel 316LV), pressure sensitive adhesive and heat-shrink tube are biocompatible and already utilized in medical devices. Therefore, no significant considerations in the biocompatibility are needed for their clinical applications. Furthermore, it is cost-effective because the raw materials for the EIS needle fabrication are inexpensive. In addition, if the manufacturing process is automated, the fabrication cost of the EIS needle can be further reduced while manufacturing reliability can be greatly improved.

The biggest problem occurring by patterning microelectrodes on the surface of biopsy needle is that parasitic impedance can be generated as shown in Fig. 3(b). This medium-dependent cell constant could be also calibrated by the method described in Fig. S4. After applying this calibration process, the composite of two media with large conductivity difference could be accurately analyzed in real-time as shown in Fig. 5(b). Calibration procedure using saline solutions with various conductivities may be a nuisance and a disadvantage in practical clinical applications. However, if proper precision manufacturing method is employed, reliable and reproducible EIS needle with consistent cell constant curves can be produced and directly used without calibration process.

## Conclusion

In this research, we proposed an EIS needle for tissue discrimination and accurate positioning of needle tip during biopsy process. By direct assembly of stainless steel microwires on the surface of biopsy needle, we could measure the electrical properties of specimen at a close vicinity of the electrode array. Especially, in our work, four electrode measurement technique was utilized for the first time in the electrical impedance measurement on a biopsy needle, in order to eliminate the electrode polarization effect and to improve the measurement accuracy. The accuracy and diagnostic performance of the EIS needle were evaluated by computational analysis. We confirmed that our EIS needle can realize highly localized impedance measurement of tissue near the microelectrode array and provide excellent diagnostic performance. Also, the capability of multi-spot sensing with multiple sets of electrode array was verified by tracking the conductivity at multiple spots. Therefore, the EIS needle was able to distinguish different types of tissues and boundary between them. This was confirmed by the experiment of hydrogel phantom with different impedance zones and porcine meat with muscle and fat tissues. Finally, EIS needle was utilized in the actual disease model such as fatty liver mouse. We observed that the measured impedance spectra by using EIS needle was similar to the previously reported differences between normal and fatty livers and verified that our EIS needle could be used to distinguish the abnormalities of lesion in real-time. Based on our experimental results, we believe that our EIS needle can be applied to conventional image-guided biopsy by enhancing the capability to detect desired lesion for tissue collection in the biopsy procedure. Especially, in case of biopsy for small size tumor, our EIS needle will be able to help the clinicians in accurate positioning of the needle tip and eventually to improve the biopsy-based early diagnosis of cancer, which will increase the complete recovery rate of the cancer patients.

## Electronic supplementary material

Supplementary information manuscript

Supplementary video_Phantom Test

Supplementary video_Porcine meat Test
