# Supplementary material for: Biopsy Needle Integrated with Electrical Impedance Sensing Microelectrode Array towards Real-time Needle Guidance and Tissue Discrimination
Source: Sci Rep. 2018 Jan 10;8:264. doi: 10.1038/s41598-017-18360-4 (PMC5762724; doi:10.1038/s41598-017-18360-4)
Supplement: Supplementary file 1 — Supplementary information manuscript [file 41598_2017_18360_MOESM1_ESM.docx]

**Electronic Supplementary Information**

**Article title:** Biopsy Needle Integrated with Electrical Impedance Sensing Microelectrode Array towards Real-time Needle Guidance and Tissue Discrimination

Jaeho Park^a^, Won-Mook Choi^b^, Kyuyoung Kim^a^, Won-Il Jeong^b^, Joon-beom Seo­­^d^ and Inkyu Park^*a,c^

^*^Corresponding author: [inkyu@kaist.ac.kr](mailto:inkyu@kaist.ac.kr), Tel: +82 42-350-3240, Fax: +82 42-350-3210

^a^Department of Mechanical Engineering, Korea Advanced Institute of Science and Technology (KAIST), Daejeon, Korea 305-701

^b^Laboratory of Liver Research, Graduate School of Medical Science and Engineering, Korea Advanced Institute of Science and Technology (KAIST), Daejeon, Korea 305-701

^c^KI for NanoCentury & Mobile Sensor and IT Convergence (MOSAIC) center, Korea Advanced Institute of Science and Technology (KAIST), Daejeon, Korea 305-701

^d^Department of Radiology, Asan Medical Center, University of Ulsan College of Medicine, Seoul, Korea


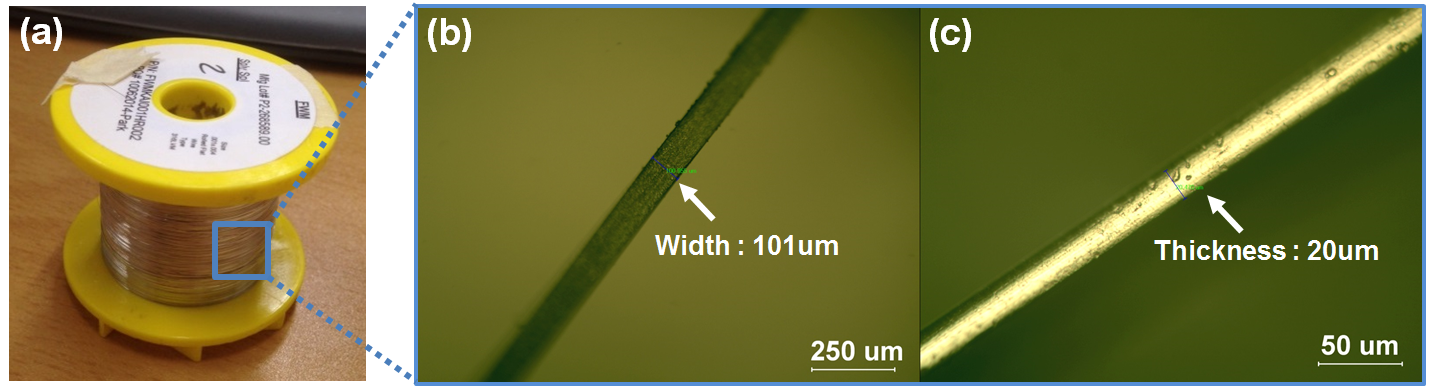


Fig. S1 Photograph and microscopic images of stainless steel flat strip used as microelectrodes in this work; The measured width and thickness of stainless steel flat strip were 101 μm and 20 μm, respectively.


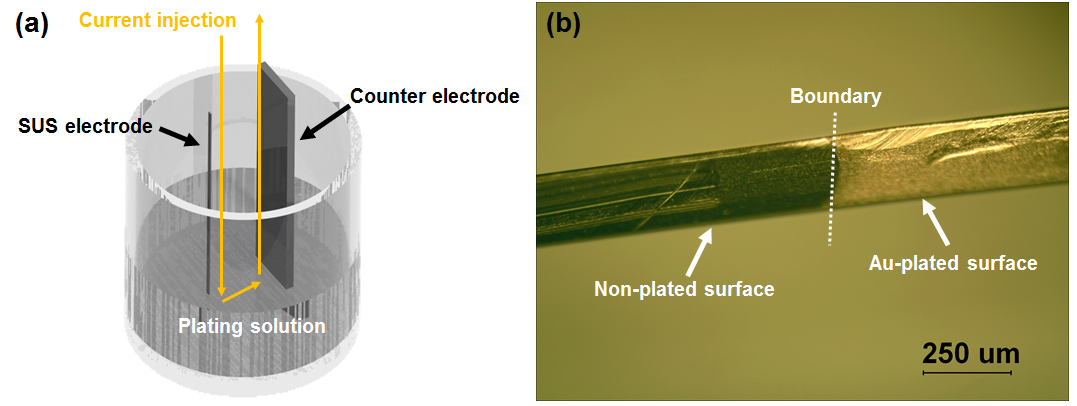


Fig. S2 Schematic image of Au electroplating process of stainless steel strip for better solderability and microscopic image of Au-plated stainless steel strip

Fig. S2 shows gold (Au) electroplating process and microscopic image of stainless steel strip after Au-plating for better solderability between the stainless steel electrodes and external wires. Because Au cannot be electroplated on the surface of stainless steel electrodes due to poor adhesion, nickel-striking was done before Au-plating. The Wood’s nickel strike solution composed of 24 g of nickel chloride (Sigma Aldrich, USA), 12.5ml of concentrated hydrochloric acid (Junsei Chemical, Japan) and 100ml of deionized water was prepared. In the Wood’s nickel strike solution, nickel was struck on the surface of stainless steel strip by injecting current with a current density of 50mA/cm^2^ for 300 sec. After the nickel strike, Au was electroplated on the surface of nickel-struck stainless steel strip. The stainless steel strip was immersed into Au-plating solution (CNC Tech., Korea) and current was injected with a current density of 1 mA/cm^2^ for 1,000 sec. As shown in Fig. S2(b), Au-layer was clearly deposited on the surface of stainless steel strip.


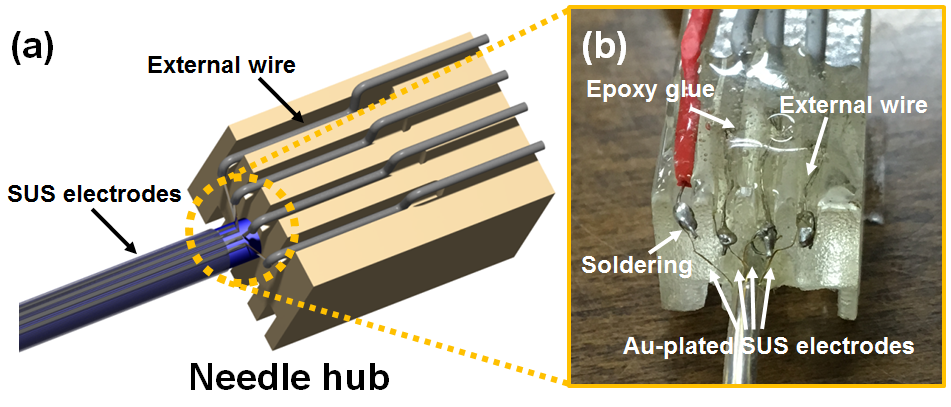


Fig. S3 Schematic and photograph image of connection between stainless steel electrodes and external wires at the needle hub.

Fig. S3 shows a schematic and photograph image of wire connection between stainless steel electrodes and external wires. After putting external wires into the trench of needle hub, external wires were further fixed with epoxy glue. The connection between stainless steel electrodes and external wires were made by conventional soldering process as shown in Fig. S3(b).

**Definition and determination of the cell constant by using saline solutions with various conductivities.**

Every probe for measuring the electrical parameters such as impedance or admittance has slightly different shapes and sizes of its electrodes, thereby the measured value is dependent on the configuration and size of used probe. Consequently, measured electrical parameters cannot tell exact electrical properties of medium without calibration process that converts into intrinsic property. Therefore, cell constant (*k*) is commonly used to convert the measured value into intrinsic property, which is independent of geometrical factor of the probe. The admittance *Y*, the reciprocal of impedance (*Z*), is described in the form of complex number as below.

$Y=\frac{1}{Z}=G+jB=G+j(2\pi fC)$ (S1)

where *Y*, *Z*, *G*, *B*, *f*, *C* and *j* are admittance, impedance, conductance, susceptance, frequency of current, capacitance and imaginary unit $\sqrt{-1}$, respectively. Also, according to Laufer et al^1^, the admittance can be described as

$Y=G+j\left( 2\pi fC \right)=k\cdot\sigma+j(2\pi f\cdot k\cdot\varepsilon_{0}\cdot\varepsilon_{r})$ (S2)

where *k*, *σ*, *ε_0_* and *ε_r_* are the cell constant of probe, conductivity, vacuum permittivity and relative permittivity of material, respectively. Since *G* and *B* can be measured with an LCR meter, the specific electric and dielectric properties of materials can be calculated if the cell constant of probe is known. A popular method for finding the cell constant is using the saline solutions since they are theoretically pure ionic conductors with constant conductivity at frequencies below 1 MHz^1^ and the relation between concentrations of NaCl and conductivities of solution is well established^2^.


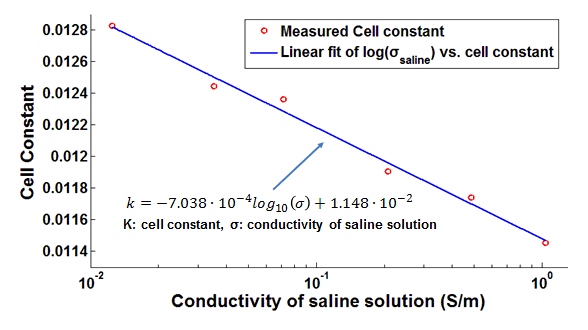


Fig. S4 Calculated cell constants in saline solutions with concentration from 0.0125 S/m to 1.037 S/m and linear fit of cell constants in log scale of saline conductivities.

Fig. S4 shows calculated cell constants in saline solutions with various conductivities from 0.0125 S/m to 1.037 S/m and linear fit graph of cell constant from conductivity change. As shown in the graph, cell constants were linearly decreased by changing the log of conductivity. The fitted curve is as follows:

*k* = -7.038·10^-4^log_10_(σ)+1.148·10^-2^ (S3)

where, *k* and σ are cell constant and conductivity of saline solution, respectively and R-square value of fitting was 0.9891. Relation between conductance, cell constant and conductivity is below.

G = *k* · σ (S4)

where G is conductance. Therefore, unknown conductivity of arbitrary saline solution can be calculated by substituting cell constant in equation S4 for modeled cell constant in equation S3 and solving equation S5 shown below. The nonlinear equation was solved by using commercial software MATLAB (MathWork^®^, USA)

G_measured_ = [-7.038·10^-4^log_10_(σ)+1.148·10^-2^]· σ (S5)

However, when different needle sample is used, coefficient of fitting model in equation S3 should be changed because arrangements of electrodes differ from each needle sample during fabrication process.


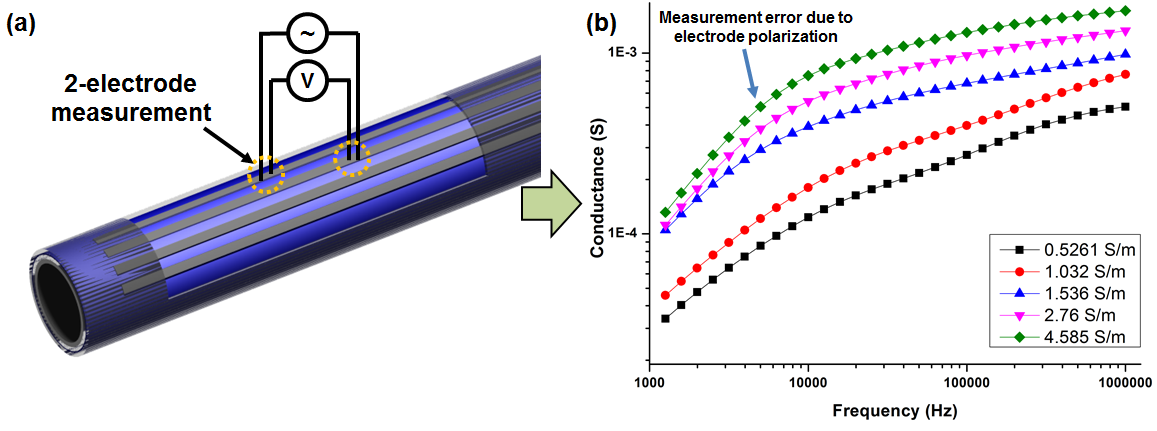


Fig. S5 Schematic image of 2-electrode measurement with fabricated EIS needle and graph of saline conductance measurement by using 2-electrode measurement method

Fig. S5(a) shows a schematic of 2-electrode measurement with fabricated EIS needle. Instead of connecting each electrode to four terminal of LCR meter, only two electrodes were connected to the terminal of LCR meter for the 2-electrode measurement method. The electrode polarization effect caused a significant measurement error at low frequency, in which the conductance of saline solution is lower than the original conductance of saline solution as shown in Fig. S5(b). The measurement errors were significantly increased at low measurement frequency because the electrode polarization induced by electrical double layer on the electrodes could not be relaxed due to the slow ion movements resulting from the slow frequency of alternative current.

**Computational analysis of tissue conductance measured by EIS needle**

Simulation was performed based on the 2D model as shown in Fig. 4(a) in manuscript in order to simplify the calculation and the depth of the model was set to 3 mm considering that the externally exposed electrode was 3 mm in actual EIS needle. The shape of EIS needle was set as a circle with a diameter of 1.2 mm, and four electrodes with a width of 110 μm, a thickness of 25 μm, and an interval of 100 μm between electrodes were attached on the surface of the needle. Diameters of cancerous tissue were varied from 1 mm to 3 mm, 5 mm, and 10 mm. The conductivities (σ) and relative permittivities (ε_r_) were set as σ=0.169 S/m and ε_r_=8·10^4^ for cancerous tissue, and σ=0.032 S/m and ε=6.6·10^4^ for normal tissue according to S. Laufer’s work^1^. A voltage of 0.1V was applied to the leftmost electrode among the four electrodes and the rightmost electrode was set to ground. At first, the needle was positioned at 8 mm away from the center of cancer tissue and the needle was moved toward center of cancer tissue with an interval of 50 μm. At each position, the conductance from voltage-carrying electrode to ground electrode was calculated.


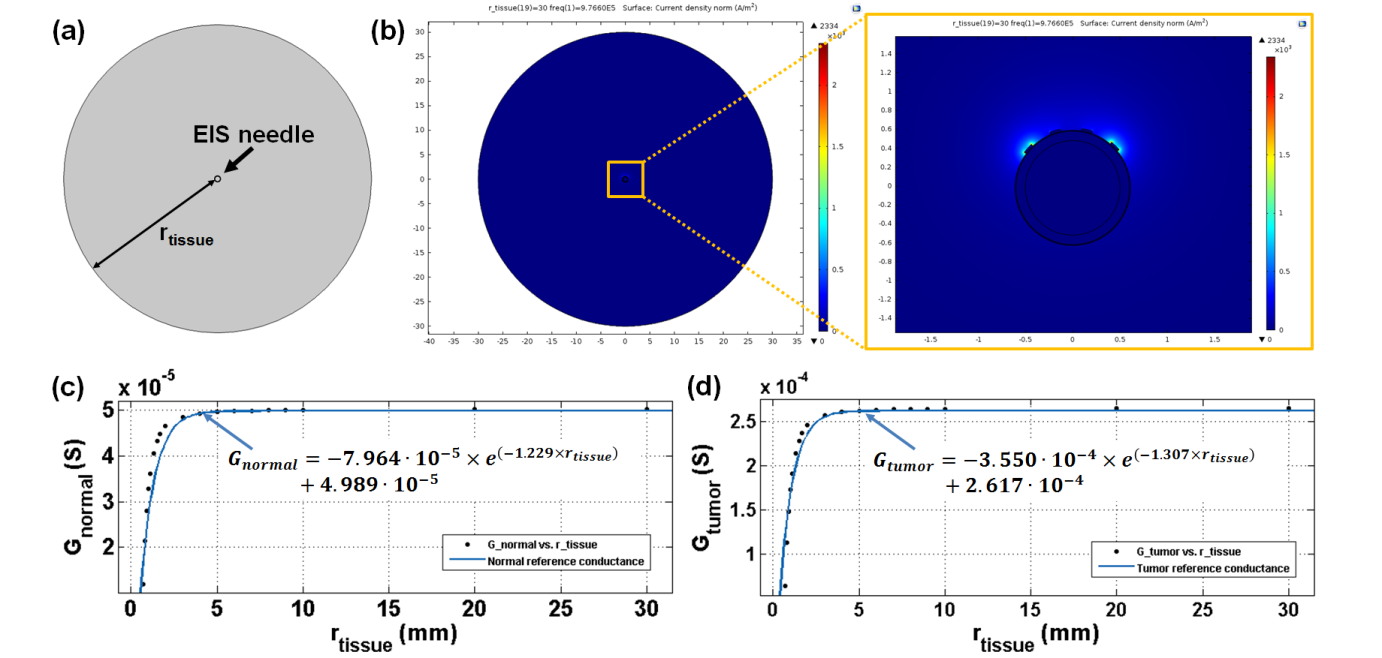


Fig. S6 Simulation model and result for calculation of reference conductance of normal and cancerous tissue: (a) image of model for reference conductance calculation; (b) current density images of overall tissue and around the EIS needle (inset); (c) result of numerical fitting for reference conductance of normal tissue; (d) result of numerical fitting for reference conductance of cancer tissue.

Fig. S6 shows the simulation model and results to obtain the reference conductances of normal and cancer tissues. We defined the reference conductance as the conductance of tissue with an infinite diameter. Therefore, the conductances of normal and tumor with radius from 0.7 mm to 30 mm was calculated by using commercial numerical simulation tool, COMSOL^®^, and the calculated conductances were numerically fitted in order to obtain the reference conductance by using a commercial numerical tool, MATLAB^®^.

As shown in Fig. S6(c-d), the reference conductances of normal tissue ($G_{pure normal})$ and cancer tissue ($G_{pure cancer})$ were calculated as 4.989·10^-5^ S and 2.617·10^-4^ S, respectively.


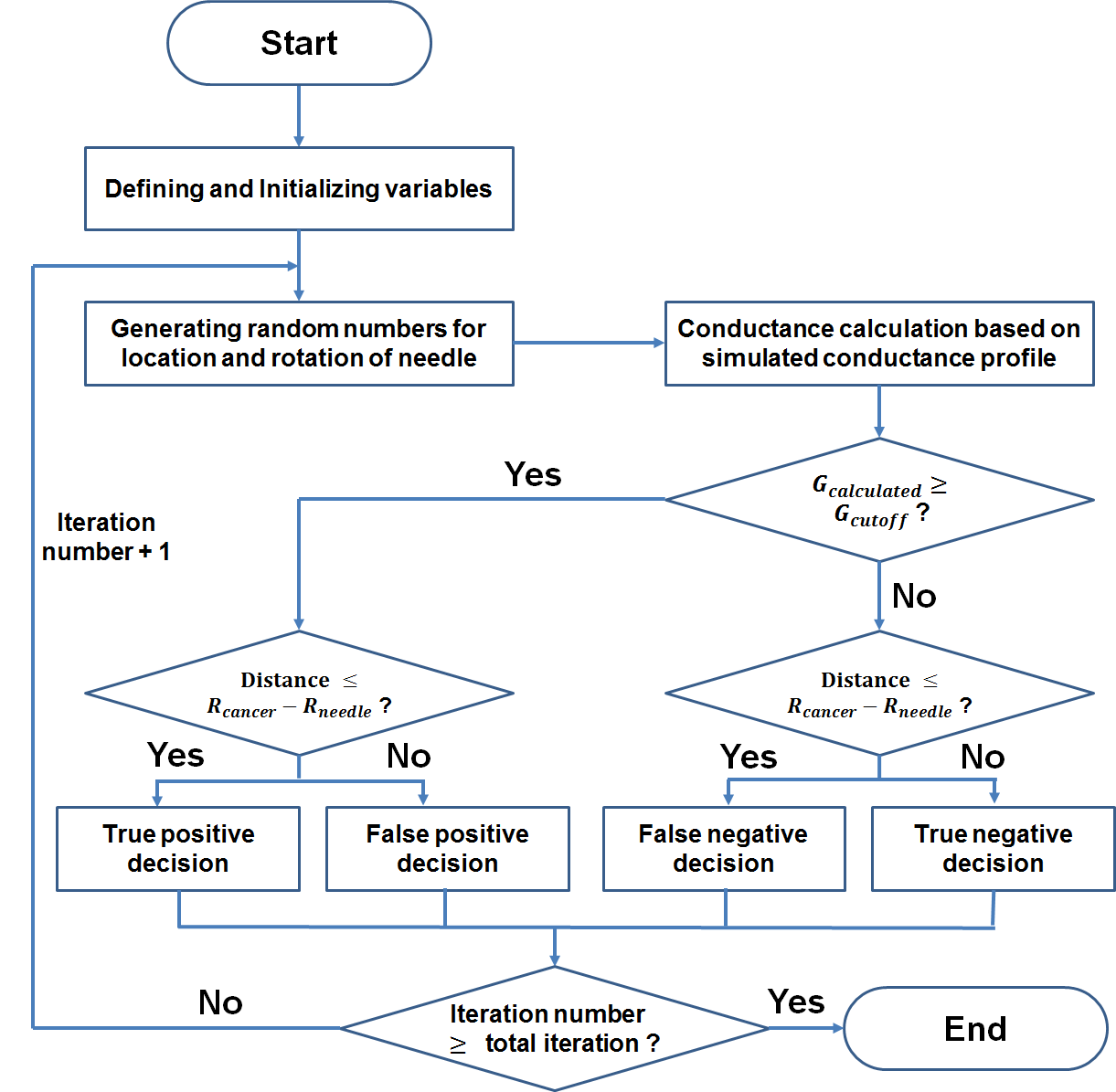


Fig. S7 Flow chart for Monte-Carlo method based simulation in order to evaluate the diagnostic performance of the proposed EIS needle.

The Monte-Carlo method based simulation was conducted in order to evaluate and obtain quantitative diagnostic performance by utilizing calculated conductance profile as shown in Fig. S8 and commercial numerical tool, MATLAB^®^.

First, after defining and initializing variables in software, the location and rotation angle of EIS needle was defined by using random number function. Based on the previously calculated conductance profile along location of needle, the conductance at defined needle location and orientation was calculated. Then, the decision was determined by using the following two criteria:

At first, when the EIS needle recognizes the cancer, the following condition should be satisfied:

Diagnosis with the EIS needle: $G_{calculated}\geq G_{cutoff}$

where *G*_cutoff_ is the cutoff conductance defined by equation 1 in the manuscript.

Also, in order to succeed the biopsy procedure, the needle should be totally inside the cancer. Therefore, below condition should be satisfied:

Success in the biopsy process: $x_{needle}$ $\leq R_{cancer}- R_{needle}$

where *x_needle_*, *R_cancer_* and *R_needle_* are location of the EIS needle center, radius of cancer and radius of needle, respectively. The location of the needle center (*x_needle_*) is defined as distance between the center of cancer and the center of the EIS needle.

Based on these two criteria, the diagnosis with the EIS needle were divided into four categories including true positive, false positive, false negative and true negative. This procedure was iterated for 5,000 times in order to obtain empirical probability of diagnostic performance with the EIS needle. Also, in reorder to plot the ROC curve of the diagnostic performance, we changed the cutoff from 0% to 90% with an interval of 1% and from 90% to 100% with an interval of 0.1%.

| Diameter of cancer | Electrode array facing toward cancer | Electrode array facing opposite  from cancer |
| --- | --- | --- |
| 1mm | 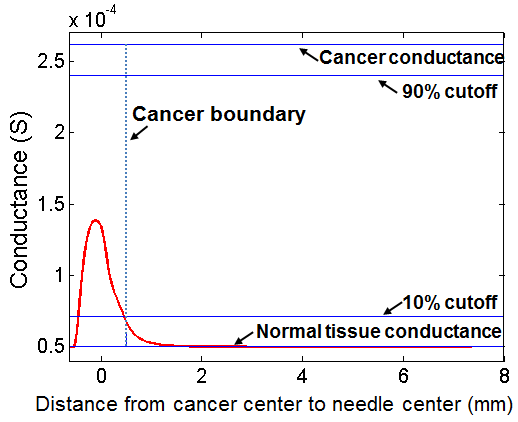 | 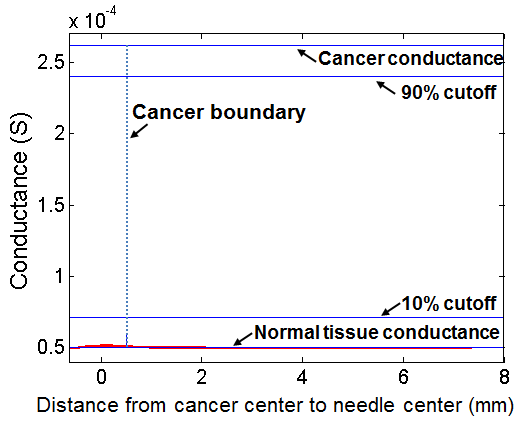 |
| 5mm | 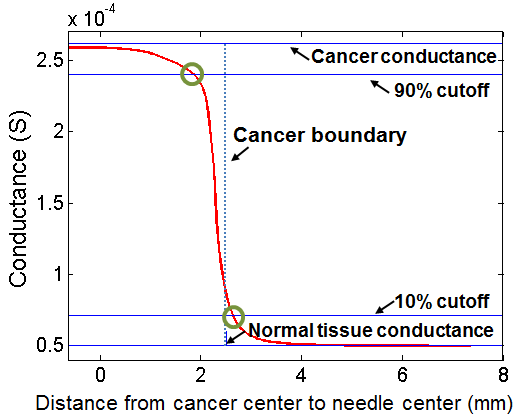 | 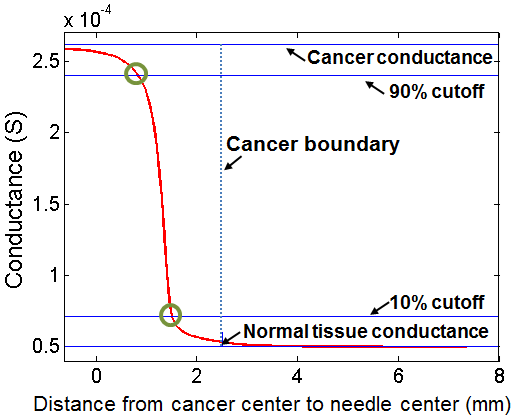 |
| 10mm | 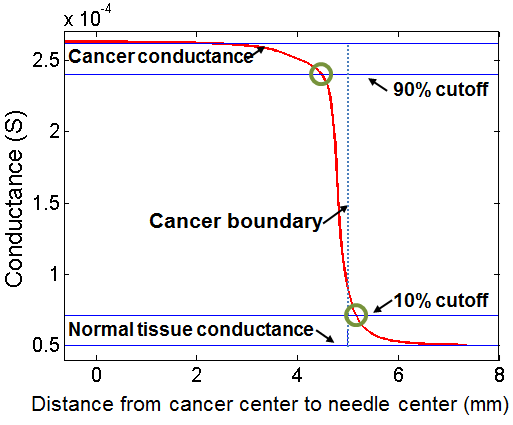 | 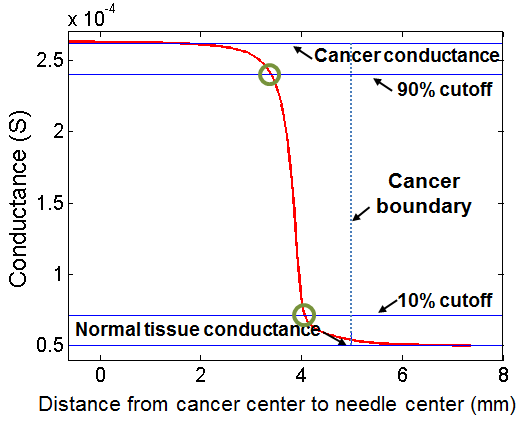 |

Fig. S8 Simulated conductance profile of the cancer-facing EIS needle (second column) and the opposite direction of EIS needle (third column) when the diameters of cancer were (a) 1mm, (b) 5mm and (c) 10mm (first column).

Fig. S8 shows the simulated conductance profile while changing the distance between the center of cancer and the center of EIS needle. Exact location of the EIS electrode, not the center of EIS needle can be calculated by subtracting 0.64 mm (radius of EIS needle) for cancer-facing EIS electrodes or by adding 0.64 mm for cancer-opposite EIS electrodes. As shown in S8(a), the conductance profile does not approach to 90% cutoff conductance because the cancer with a diameter of 1 mm is too small while the diameter of EIS needle is 1.28 mm. For cancer diameter of 5 mm and 10 mm, calculated location of the EIS needle and distance between the center of EIS needle and cancer boundary for 10% and 90% cutoff conductances are summarized in Table S1.

**Table S1 Calculated locations of EIS needle and distance between the center of EIS needle and cancer boundary for EIS electrodes facing towards cancer or in the opposite direction to the cancer tissue (diameter = 5 mm and 10 mm).**

|  | | EIS electrodes facing toward cancer | | EIS electrodes facing in the opposite direction to cancer | |
| --- | --- | --- | --- | --- | --- |
| Cancer diameter | Cutoff  percentage | Location of EIS needle | Distance between needle center and cancer boundary | Location of EIS needle | Distance between needle center and cancer boundary |
| 5 mm | 10% | 3.281 mm | 0.781 mm | 2.156 mm | 0.344 mm |
|  | 90% | 2.501 mm | 0.001 mm | 1.477 mm | 1.023 mm |
| 10 mm | 10% | 5.821 mm | 0.821 mm | 4.719 mm | 0.281 mm |
|  | 90% | 5.126 mm | 0.126 mm | 4.041 mm | 0.959 mm |


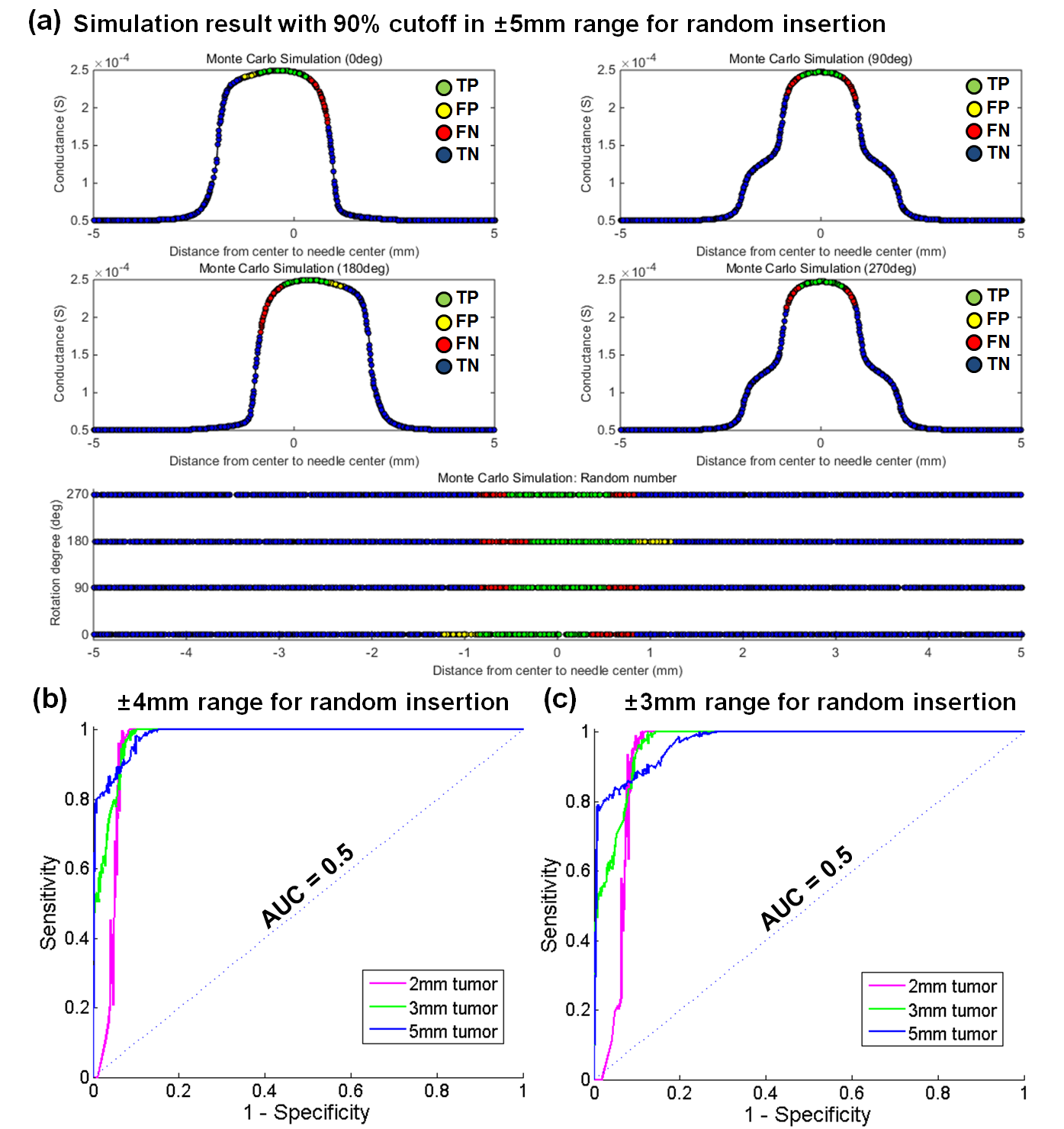


Fig. S9 Result of Monte-Carlo method based simulation for diagnostic performance of the EIS needle: (a) the simulation result with 3 mm diameter cancer and 90% cutoff in ±5 mm range of random insertion. The graphs in the first and second rows are the conductance profiles that depict the decision at the needle locations. The green, yellow, red and dark blue colors mean the true positive (TP), false positive (FP), false negative (FN) and true negative (TN), respectively. The third row shows the randomly generated locations and rotation angles with the decision dots; (b) receiver operating characteristic curve for random insertion of ±4 mm range and (c) receiver operating characteristic curve for random insertion of ±3 mm range

Fig. S9(a) shows the result of Monte-Carlo method based simulation for cancer with diameter of 3 mm and 90% cutoff conductance by the EIS needle. The range of random insertion was set to ±5 mm, which assumed that the clinicians cannot position the biopsy needle more accurately than 10 mm range. When the needle is located at the center of cancer, the EIS needle can recognize the cancer conductance and the biopsy is also successful because the needle is entirely in the cancer region (i.e. true positive). However, when the needle is moved toward outside of cancer, misdiagnosis starts to occur as depicted by yellow (false positive) and red (false negative) dots.

In case of the receiver operating characteristic (ROC) curve shown in Fig. 5(d), the range of random insertion was ±5 mm (total 10 mm). However, if clinicians have more advanced biopsy skills with higher positioning accuracy, range of random insertion would be reduced and this the probability of true negative would decrease. Therefore, the ROC curve with the range of random insertion of ±4 mm (total 8 mm), ±3 mm (total 6 mm) was simulated as shown in Fig. S9(b-c). As shown in graph, the ROC moved from the perfect identifier condition at (0, 1) to random identifier condition (1-Specificity = Sensitivity line). This was because the specificity became larger as defined in equation (4) and rate of misdiagnosis to total diagnosis become larger. Although the diagnostic performance is poorer for the range of random insertion of ±4 mm (total 8 mm), ±3 mm (total 6 mm) than that for the range of random insertion of ± 5mm, all of area under ROC curve (AUC) is larger than 0.9 (see Table S2). Therefore, the EIS needle based biopsy process can be useful for accurate needle positioning in conventional image-guided biopsy process.

**Table S2 Calculated area under ROC curve for cancers with diameter of 2 mm, 3 mm and 5 mm under ±4 mm and ±3 mm range of random insertion**

| Cancer diameter | ±4 mm range | ±3 mm range |
| --- | --- | --- |
| 2 mm | 0.952 | 0.934 |
| 3 mm | 0.978 | 0.967 |
| 5 mm | 0.984 | 0.971 |


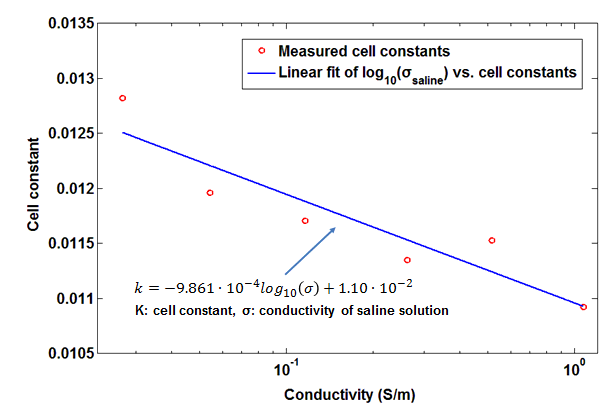


Fig. S10 Calculated cell constants of the EIS needle used in phantom test and linear fit of cell constant vs. log of saline conductivities.

Fig. S10 shows calculated cell constants of the EIS needle used in phantom test based on various saline solutions with concentrations from 0.027 S/m to 1.074 S/m in order to calibrate effect from parasitic component of the fabricated EIS needle. When the cell constant was fitted to a linear function of the log of saline conductivity, calculated equation of the cell constant was obtained as follows:

*k* = -9.861·10^-4^log_10_(σ)+1.10·10^-2^ (S6)

Then unknown conductivity measured at the needle tip could be calculated by solving the following equation with known measured conductance from the LCR meter.

G_measured_ = [-9.861·10^-4^log_10_(σ)+1.10·10^-2^]· σ (S7)


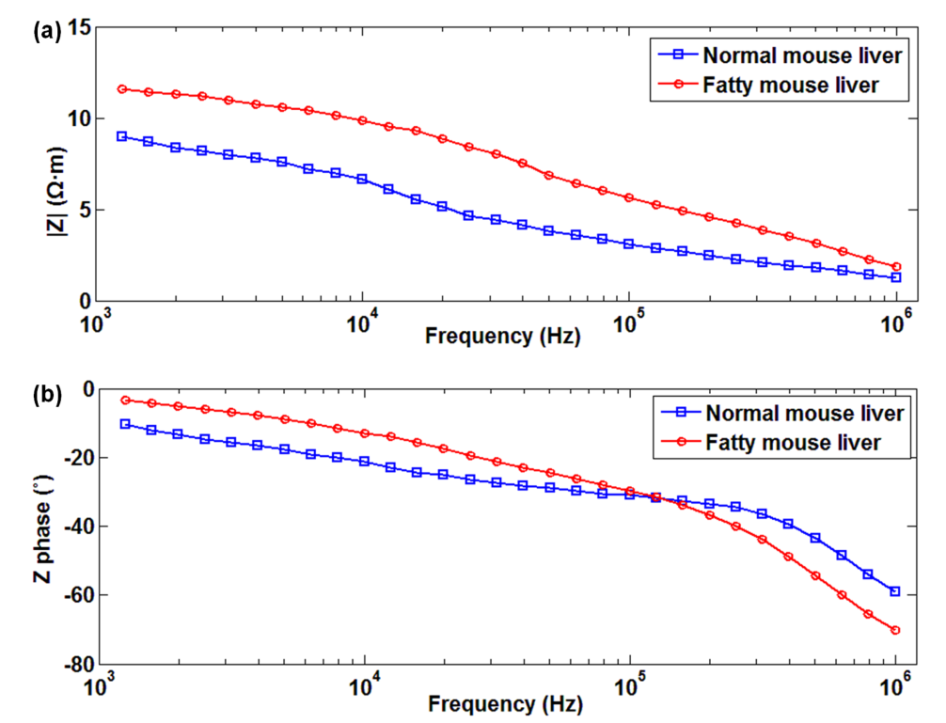


Fig. S11 Measured impedance spectra and phase angle from normal and fatty mouse liver at frequencies from 1 kHz to 1 MHz

Fig. S11 shows the impedance and phase angle values from normal and fatty mouse liver measured by the fabricated EIS needle. Impedance values were calculated by reciprocals of measured complex admittances from LCR meter and phase angle was calculated by an arc tangent of the real part over the imaginary part of complex impedance. The measured impedances and phase angle of normal and fatty livers at 1 kHz, 10 kHz, 100 kHz and 1MHz frequency are summarized in Table S3.

**Table S3 Measured values of impedance and phase angle of normal and fatty mouse livers at frequencies of 1 kHz, 10 kHz, 100 kHz and 1 MHz**

|  | Parameters | 1 kHz | 10 kHz | 100 kHz | 1 MHz |
| --- | --- | --- | --- | --- | --- |
| Normal mouse liver | Impedance (Ω·m) | 8.985 | 6.665 | 3.076 | 1.232 |
|  | Phase angle (˚) | -10.33 | -21.31 | -30.9 | -59.02 |
| Fatty mouse liver | Impedance (Ω·m) | 11.56 | 9.855 | 5.626 | 1.849 |
|  | Phase angle (˚) | -3.514 | -12.94 | -29.61 | -70.1 |

**The Monte-Carlo simulation result viewer has large file size.**

**Therefore, instead of direct upload of files, we uploaded the URL of result viewer program which was uploaded in Google Drive**

**URL for download: https://goo.gl/tobvzs**

**References**

1 Laufer, S., Ivorra, A., Reuter, V. E., Rubinsky, B. & Solomon, S. B. Electrical impedance characterization of normal and cancerous human hepatic tissue. *Physiol Meas* **31**, 995-1009, doi:10.1088/0967-3334/31/7/009 (2010).

2 Peyman, A., Gabriel, C. & Grant, E. H. Complex permittivity of sodium chloride solutions at microwave frequencies. *Bioelectromagnetics* **28**, 264-274, doi:10.1002/bem.20271 (2007).
